# Association between polymorphisms of the adenylate cyclase 3 gene rs2241759 and the effect of high-intensity interval training on blood lipid profiles

**DOI:** 10.7717/peerj.19271

**Published:** 2025-04-11

**Authors:** Junren Lai, Li Gong, Yan Liu, Yanchun Li, Jing Ni, Duoqi Zhou

**Affiliations:** 1 College of Life Science, Anqing Normal University, Anqing, China; 2 College of Sport, Anqing Normal University, Anqing, China; 3 Scientific Research Center of Chinese Sports and Health, Beijing Sport University, Beijing, China; 4 College of Sport, JiangXi Normal University, Nanchang, Jiangxi, China

**Keywords:** Adenylate cyclase 3, Gene polymorphisms, High-intensity interval training, Blood lipid profiles

## Abstract

**Background:**

One of the recognized effects of systematic physical activity is the improvement of physical fitness, with a negative correlation found between physical fitness and cardiovascular and cardiometabolic risk. The purpose of this study is to analyze the influence of single nucleotide polymorphisms (SNPs) of the adenylate cyclase 3 (*ADCY3*) gene on the effect of high-intensity interval training (HIIT) on blood lipids, and simultaneously screen out the genetic markers sensitive to HIIT in Chinese Han youth.

**Methods:**

In the 12-week HIIT program, a total of 237 Chinese Han college students with non-regular exercise habits were recruited, and these volunteers participated in the training three times a week. Baseline and after the HIIT program, total cholesterol (TC), triglyceride (TG), high-density lipoprotein cholesterol (HDL-C) and low-density lipoprotein cholesterol (LDL-C) were measured, respectively. DNA was extracted from the white blood cells of volunteers and genotyping was carried out. The PLINK v1.09 software was used to conduct quality control screening on the obtained SNPs, and a linear regression model was constructed to analyze the association between *ADCY3* gene SNPs and the effect of HIIT on blood lipids. ANOVA multiple comparison (LSD) was performed to test the difference between groups (*P* < 0.05).

**Results:**

(1) Through the analysis of Illumina CGA chip scanning, a total of 22 SNPs of the *ADCY3* gene were identified. Following rigorous quality control screening, 15 SNPs were included in the subsequent analysis. Notably, it was found that the rs2241759 locus is associated with the effect of HIIT on blood lipid profiles. (2) Among male volunteers, significant differences in the baseline HDL-C values were observed among the three genotypes at the rs2241759 locus of the *ADCY3* gene (β =  − 0.019, *P* = 0.040). The baseline value for the GG genotype was higher than that AA/AG genotypes. (3) After HIIT, the total levels of TC and HDL-C in volunteers increased significantly (*P* < 0.05). In contrast, the total levels of TG and LDL-C decreased significantly (*P* < 0.05). Further statistical analysis categorized by gender revealed that, with the exception of TC values in men, significant changes were observed for TC, TG, HDL-C, and LDL-C across both genders (*P* < 0.05). (4) Compared to male volunteers with the GG genotype, male volunteers carrying the A allele exhibited a more pronounced change in TC values following training (β = 0.044, *P* = 0.038). (5) The rs2241759 locus demonstrated a significant association with the effect of HIIT on LDL-C (β =  − 0.065, *P* = 0.04363).

**Conclusion:**

(1) The implementation of a 12-week HIIT regimen can significantly enhance the blood lipid status of college students. (2) The locus rs2241759 of the *ADCY3* gene is significantly associated with the sensitivity of LDL-C to HIIT.

## Introduction

Blood lipids refer to total cholesterol (TC), high-density lipoprotein cholesterol (HDL-C), low-density lipoprotein cholesterol (LDL-C), and triglycerides (TG) present in serum. LDL-C and TG are closely associated with obesity, atherosclerosis, type 2 diabetes mellitus (T2DM), hypertension, and other related diseases (Bauersachs et al., 2019). According to Chinese Guidelines for Lipid Management (2023), since the 1980s, blood lipid levels among Chinese citizens—including children and adolescents—have undergone significant changes, leading to a marked increase in the incidence of dyslipidemia. Furthermore, there is a notable lack of awareness regarding dyslipidemia among Chinese residents, resulting in relatively low rates of prevention and treatment. The current state of preventive measures and treatment efforts is concerning.

Data indicate that the insufficient annual average effective exercise time among Chinese citizens contributes, either directly or indirectly, to an increase in the average levels of LDL-C, TC, and TG, while simultaneously leading to a decrease in HDL-C values (Liang, Ke & Liu, 2024). Exercise is an effective method to prevent/treat dyslipidemia, particularly through the efficient utilization of exercise programs and time allocated by schools. Such interventions can significantly mitigate obesity and coronary artery disease (CAD) in adolescents (Liu et al., 2024; Wang & Ye, 2024). A study has demonstrated that both moderate-intensity continuous training (MICT) and HIIT can substantially reduce body weight and body fat while significantly enhancing maximal oxygen uptake (VO2max); however, the improvements observed with HIIT were more pronounced. Additionally, it was found that only volunteers in the HIIT group exhibited a significant increase in their maximum fat oxidation rate (Vaccari et al., 2020). Another investigation revealed that HIIT may be a time-efficient intervention for counteracting dyslipidemia (Alvarez et al., 2018). Consequently, customized HIIT programs have emerged as a predominant approach within health promotion interventions in recent years. Nevertheless, individuals display considerable variability regarding exercise effectiveness. A silencer region located within intron 16 of the gene encoding angiotensin-converting enzyme (ACE), which regulates vascular perfusion, has been implicated in phenotypic variations related to aerobic fitness and susceptibility to type 2 diabetes mellitus (T2DM) (Flück et al., 2022). Following power-matched one-legged cycling exercises, non-carriers of the T-allele for monocarboxylate transporter-1 gene (MCT1) demonstrated diminished lipid handling capabilities (Gasser et al., 2024). Thus, genetic factors may partially elucidate individual differences observed in training effectiveness (Bouchard et al., 2015). Previous studies have demonstrated that after 16-week of aerobic training, the LDL-C levels increased in carriers of the rs693 SNP of the *ApoB* gene (Tamburus et al., 2018). The D allele of MDM4 rs35493922 I/D was found to be over-represented in power athletes compared to controls (*P* = 7.8 ×10^−9^) and endurance athletes (*P* = 0.0012). Furthermore, this D allele exhibited a positive association (*P* = 0.0013) with greater fat-free mass in the UK Biobank cohort (Kazan et al., 2024). These findings suggest a significant relationship between gene polymorphisms and the effect of HIIT on blood lipid profiles.

The adenylate cyclase 3 (*ADCY3*) gene, which encodes for the enzyme ADCY3, is regulated by G-proteins and catalyzes the conversion of adenosine triphosphate (ATP) to produce 3′, 5′-cyclic adenosine monophosphate (cAMP). ADCY3 is implicated in lipid metabolism and olfactory functions and participates in various signaling pathways, including those involving Ca^2+^/calmodulin as well as adrenergic receptors (Fitzpatrick & Solberg Woods, 2024; Peng et al., 2023; Sadana & Dessauer, 2009). Research indicates that multiple signaling pathways associated with ADCY3 may be related to ischemic stroke resulting from atherosclerosis (Zhang, Guo & Zhou, 2023). In obese individuals treated with glucagon-like peptide-1 (GLP-1), significant decreases were observed in weight, body mass index (BMI), and waist-to-hip fat ratio. Conversely, levels of ADCY3, HDL-C, and TG showed marked increases following treatment (Li et al., 2017). Thus, it is suggested that the *ADCY3* gene may contribute to improvements in blood lipid profiles.

In summary, the polymorphisms of the *ADCY3* gene are speculated to be associated with the effect of HIIT on blood lipid profiles, which may exhibit sensitivity to HIIT. However, there are limited research reports on this topic. Consequently, this study focused on the *ADCY3* gene and examined the changes in blood lipid profiles among volunteers with different genetic genotypes over a 12-week HIIT program. The aim was to explore whether *ADCY3* gene polymorphisms and blood lipid profiles show sensitivity to HIIT. This research seeks to provide molecular markers that can aid in developing personalized exercise plans aimed at improving blood lipid status, as well as offering a theoretical foundation for preventing and treating dyslipidemia-related diseases through exercise interventions.

## Materials & Methods

### Participants

A total of 259 Chinese Han university students with non-regular exercise were recruited from Anqing Normal University, Jiangxi Normal University, Inner Mongolia Normal University, and Lanzhou City College at first. After conducting a quality control screening, a total of 237 volunteers were ultimately included in the study (Table 1). (The criteria for screening and the reasons for exclusions are described in detail below). Beijing Sport University has taken charge of this experiment and offered professional technical guidance. Volunteers were required to be physically healthy, have no family history of hereditary diseases, have no chronic diseases, and have not taken high-dose antibiotics, steroid drugs, or opioids within 3 months. Before the experiment, each volunteer signed an informed consent form and was informed of the experimental risks; Each volunteer was required to fill out the “Exercise Risk Screening Questionnaire” (including health survey and physical activity questionnaire, File S3), and researchers would conduct exercise risk assessments on the volunteers based on this survey to ensure the safety and effectiveness of the experiment. During the experiment, volunteers were required to have a normal daily routine, diet, not take the aforementioned drugs, and not engaged in any other exercise interventions. The experimental plan has been approved by the Ethics Committee of Beijing Sport University, with an ethics review approval number of 2018018H.

**Table 1:** Mean value ± standard deviation (SD) of the volunteers basic information.

Variable	*n*	Age/year	Height/cm	Weight/kg	BMI (kg/m^2^)
Total	237	21.00 ± 1.11	166.37 ± 8.67	59.56 ± 11.86	21.21 ± 2.98
Male	109	21.00 ± 1.11	166.37 ± 8.67	67.16 ± 11.86	21.20 ± 2.97
Female	128	20.94 ± 1.06	165.66 ± 8.77	53.08 ± 12.25	21.26 ± 3.05
*P*	–	0.628	0.321	0.768	0.221

**Notes.**

* *P* < 0.05.

** *P* < 0.01.

### Training protocol

Volunteers were instructed to refrain from engaging in moderate- and high- intensity exercise, maintain a regular diet, and adhere to normal work-rest schedules for three days prior to the test. The VO2max test was performed using a power cycling incremental load exercise protocol, with direct measurements obtained from the COR-TEX gas metabolism analyzer (Cortex MetaMax 3B, Leipzig, Germany). Test parameters: The initial workload for male volunteers was set at 50 W, increasing by 25 W/2 min. For female volunteers, the initial workload started at 40 W, with increments of 20 W/2 min. The pedaling frequency was controlled to achieve a consistent rate of 60 rpm using a metronome. Throughout the exercise, real-time heart rates of the volunteers were monitored using a Polar heart rate strap (Polar China Co., Ltd, Guangzhou, CN), and the ratings of perceived exertion (RPE) for each volunteer were recorded at every load level. Dynamic electrocardiogram monitoring was implemented throughout the testing process to assess potential exercise-related risks. When at least 2 of the following 4 conditions are satisfied, it is determined as V̇O2max: (1) The respiratory quotient reaches 1.1; (2) Heart rate exceeds 90% of the predicted maximum heart rate (HRmax); (3) Oxygen uptake plateaus and no longer increases with increasing load; 4) The volunteers’ RPE ≥ 17 and cannot complete the current load. Record the absolute value of V̇O2max, as well as the VEmax, HRmax, RER, CO2max, and calculate the relative value of V̇O2max by dividing the absolute value by the volunteers’ test weight. The training intensity was set according to the VO2max. A HIIT mode was adopted. The training was carried out 3 times a week for 12-week. The intensity of high-intensity running was 80%–90% of VO2max, and the interval intensity was 50%–55% of VO2max (Table 2). By wearing the Polar heart-rate strap, the heart rate of the volunteers was controlled within the heart-rate range corresponding to the target intensity. The four recruited universities each provided training venues (the training venue was either an outdoor or indoor track-and-field, and if the weather conditions limit, a treadmill will be used for the test) and completed all training contents and index tests during the period from September to December in 2020. Each training session was guided by a professional training team to ensure the safety and effectiveness of the training.

**Table 2:** Training plan of HIIT.

Training phase	Weeks	Plan	Intensity	Time
Adaptive phase	Week 1	Running: 56 sets× 15s	80–90%VO2max (Heart rate:180–190 ± 5 beats\min)	28 min\time 3 times\week
		Walking: 56 sets× 15s	50–55% VO2max (Heart rate:140 ± 6 beats\min)	
	Week 2	Running: 28 sets× 30s	80–90%VO2max (Heart rate:180–190 ± 5 beats\min)	28 min\time 3 times\week
		Walking: 28 sets× 30s	50–55% VO2max (Heart rate:140 ± 6 beats\min)	
	Week 3	Running: 14 sets× 1min	80–90%VO2max (Heart rate:180–190 ± 5 beats\min)	28min\time 3 times\week
		Walking: 14 sets× 1min	50–55% VO2max (Heart rate:140 ± 6 beats\min)	
	Week 4	Running: 7 sets× 2min	80–90%VO2max (Heart rate:180–190 ± 5 beats\min)	28min\time 3 times\week
		Walking: 7 sets× 2 min	50–55% VO2max (Heart rate:140 ± 6 beats\min)	
Improvement phase	Week 5–12	Running: 4 sets× 4min	80–90%VO2max (Heart rate:180–190 ± 5 beats\min)	28 min\time 3 times\week
		Walking: 4 sets × 3 min	50–55% VO2max (Heart rate:140 ± 6 beats\min)	

### Blood lipids and body composition testing

Fasting for a duration of 10 h prior to the initial training session. In order to mitigate any acute effects of exercise, following a period of 72 h after the last training session, and then fast for another 10 h. Five ml of venous blood from each volunteer was drawn to test blood lipid indicators, including total cholesterol (TC), high-density lipoprotein cholesterol (HDL-C), low-density lipoprotein cholesterol (LDL-C), and triglycerides (TG). The testing instrument is the Beckman DXC800 (Beckman Coulter. Inc, Brea, CA, USA) fully automatic biochemical analysis system. All volunteers were subjected to blood sampling and testing by professional personnel. Body composition was testing by Inbody 260 (InBody Co., Ltd, Shenzhen, China), including height, weight, BMI, *etc*.

### Gene extraction and typing

Extract the DNA of white blood cells from the blood of volunteers by using a DNA extraction kit (Tiangen Biochemical Technology Co., Ltd, Beijing, China). Use the Infinium chip (chip type: CGA 24v1-0, Illumina (Illumina Co., Ltd, San Diego, CA, USA)) to perform whole-genome genotyping, and a total of 259 people were successfully genotyped. Extract the autosomal genotype data from the whole-genome data of 259 people for data quality control. Quality control conditions (Purcell et al., 2007): eliminate the SNPs with a genotype data missing rate >3% (52,874 SNPs), eliminate the samples with a detection rate <90% (10 people), eliminate the SNPs with a minor allele frequency (MAF) <1% (149,912 SNPs), eliminate the SNPs that do not conform to the Hardy-Weinberg equilibrium (*P* <  1 × 10^−^^6^, 545 SNPs), eliminate the samples with a heterogeneity test deviation from the “Mean 3SD” (five people), and eliminate the samples with a PCA analysis deviation from the “Mean 3SD” (seven people). A total of 237 people and 485,474 SNPs of the original chip genotyping data passed the quality control. 22 SNPs of *ADCY3* gene were ultimately screened out.

### Statistical analysis

SPSS 26.0 software (SPSS Inc., Armonk, NY, USA) was used for performing normal distribution test on data of blood lipids, paired sample *T*-test to detect changes in blood lipids value before and after training. Quality control screening on the SNPs was conducted using the PLINK v1.09 software, with the standard being: MAF >0.05; SNPs call rate >90% (Tchio et al., 2021); H-W *P* > 0.05, and then further correlation analysis was performed between the screened SNPs and the effect of HIIT on blood lipid profiles. Using the age, gender, and baseline blood lipid values as covariates (*i.e.,* eliminating the influence of age, gender and other factors on phenotype), a linear regression model was constructed using PLINK v1.09 software. Under the influence of the additive effects model (ADD), correlation analysis was conducted between the selected SNPs genotypes and different phenotypes. The multiple comparison (LSD) one-way analysis of variance (ANOVA) tests for comparing the differences in variables conforming to a normal distribution between groups, with a significance level of 0.05. The mean ± SD (standard deviation) was employed to report continuous variables.

The study was performed using a linear regression model to evaluate the genotypes for additive effects

\begin{eqnarray*}Y=Xb+Z\alpha +. \end{eqnarray*}

The vector (*Y*) represents phenotypic observations, while (*X*) denotes the incidence matrix that correlates phenotypes with fixed effects such as age, gender, and baseline blood lipid levels. The vector of fixed effects is denoted by (b). The matrix (Z) corresponds to the genotypic incidences (coded as 0 for the first homozygote AA; 1 for the heterozygote AG; and 2 for the second homozygote GG) of the analyzed SNPs. Additionally, (alpha) symbolizes the vector of regression coefficients associated with SNP effects, whereas (*ɛ*) indicates the vector of residual effects assumed to follow a normal distribution: (N ∼ (0, I*σ*^2^_e_)), where (*σ*^2^_e_) represents the residual variance. A Bonferroni-adjusted threshold of 0.05 was used to allow for hypothesis testing using 0.05/N, where N is the number of SNPs analyzed.

## Results

### SNPs of *ADCY3* gene

**Figure 1:**
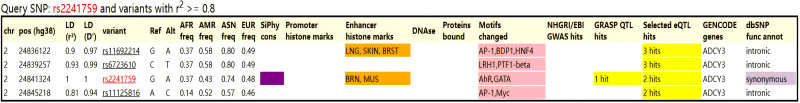
Co-inheritance map of the rs2241759 locus in the ADCY3 gene.

After scanning with Illumina CGA gene chip, a total of 22 SNPs was obtained, and 15 SNPs that met the standards were obtained after quality control screening using PLINK software (Table 3). No co-inheritance SNPs were found with rs2241759 using HaploReg v.4.2 (https://pubs.broadinstitute.org/mammals/haploreg/haploreg.php), so we conducted further analysis on rs2241759 locus (Fig. 1).

**Table 3:** Information of the *ADCY3* gene SNPs included in the study after quality control.

CHR	SNP	Location	Major allele	Minor allele	MAF	Genotype counts	H-W *P* value
2	rs1056074	25,053,828	C	T	0.253	16/89/132	1.0
2	rs6753096	25,055,630	C	T	0.291	23/92/122	0.4258
2	rs4665730	25,055,774	G	A	0.386	34/118/85	0.4583
2	rs71439148	25,077,569	G	A	0.086	4/33/200	0.1605
2	rs1541984	25,082,414	A	G	0.466	51/119/67	1.0
2	rs11687089	25,082,926	C	T	0.466	53/119/65	1.0
2	rs13407913	25,097,644	G	A	0.462	50/119/68	0.497
2	rs13410999	25,097,939	C	T	0.466	50/121/66	0.8152
2	rs2033654	25,103,108	A	C	0.46	39/120/78	1.0
***2***	rs2241759 ^*^	***25,064,193***	***G***	***A***	***0.281***	**16/102/119**	***0.511***
2	rs6725517	25,129,473	G	A	0.451	48/118/71	1.0
2	rs6545814	25,131,316	G	A	0.422	41/118/78	0.8797
2	rs2384061	25,135,620	A	G	0.418	43/114/80	1.0
2	rs10203386	25,136,866	A	T	0.424	44/113/80	0.7883
2	rs11676272	25,141,538	G	A	0.424	42/117/78	1.0

**Notes.**

* Bold italics indicate significant differences, *P* <  0.05.

### Association between *ADCY3* gene polymorphisms and baseline blood lipid profiles

In total levels, the values of TC, LDL-C and TG in individuals with AA/AG genotypes were higher than those in individuals with GG genotype, while the value of HDL-C in individuals with GG genotype was higher than that in individuals with AA/AG genotypes. However, none of the changes were statistically different (*P* > 0.05). The HDL-C value in male individuals with GG genotype was significantly higher than that in those with AA/AG genotypes (β = −0.019, *P* = 0.040) (Table 4).

**Table 4:** Associations between *ADCY3* gene rs2241759 polymorphism and the baseline blood lipid profiles.

Blood lipids	Variable	*n*	AA (mmol\L)	AG (mmol\L)	GG (mmol\L)	Major allele	TEST	*β*	*P*
TC	Total	237	3.978 ± 0.640	3.990 ± 0.633	3.953 ± 0.630	G	ADD	0.044	0.419
	Male	109	3.978 ± 0.638	3.984 ± 0.637	3.943 ± 0.626	G	ADD	−0.048	0.071
	Female	128	4.008 ± 0.615	4.009 ± 0.616	3.979 ± 0.622	G	ADD	0.017	0.415
HDL-C	Total	237	1.505 ± 0.364	1.517 ± 0.361	1.528 ± 0.367	G	ADD	−0.018	0.726
	Male	109	1.505 ± 0.363	1.514 ± 0.362	1.525 ± 0.370	G	ADD	−0.019	0.040^*^
	Female	128	1.541 ± 0.358	1.541 ± 0.359	1.565 ± 0.364	G	ADD	0.033	0.288
LDL-C	Total	237	2.400 ± 0.455	2.404 ± 0.453	2.384 ± 0.450	G	ADD	0.948	0.430
	Male	109	2.400 ± 0.455	2.398 ± 0.455	2.381 ± 0.450	G	ADD	0.491	0.232
	Female	128	2.409 ± 0.447	2.410 ± 0.446	2.391 ± 0.452	G	ADD	−0.015	0.368
TG	Total	237	1.233 ± 0.444	1.233 ± 0.446	1.222 ± 0.436	G	ADD	−0.030	0.989
	Male	109	1.233 ± 0.444	1.234 ± 0.449	1.222 ± 0.436	G	ADD	0.014	0.347
	Female	128	1.218 ± 0.431	1.217 ± 0.432	1.214 ± 0.457	G	ADD	−0.065	0.105

**Notes.**

* *P* < 0.05.

TC total cholesterol HDL-C high-density lipoprotein cholesterol LDL-C low-density lipoprotein cholesterol TG triglycerides

### The effect of HIIT on blood lipid profiles

After 12-week of HIIT, the total values of TC and HDL-C of the volunteers significantly increased (*P* <  0.01), while the total values of LDL-C and TG significantly decreased (*P* <  0.01). Further gender analysis revealed that there were no statistically significant training changes in male TC (*P* >  0.05), but total values of HDL-C, LDL-C, TC, and TG showed significant change (*P* < 0.01) (Table 5).

**Table 5:** The effect of HIIT on blood lipid profiles.

Blood lipids	Variable	*n*	Δ(mmol/L)	*P*
TC	Total	237	0.108 ± 0.535	0.002^**^
	Male	109	0.101 ± 0.535	0.413
	Female	128	0.116 ± 0.513	0.001^**^
HDL-C	Total	237	0.276 ± 0.251	0.000^**^
	Male	109	0.280 ± 0.251	0.000^**^
	Female	128	0.259 ± 0.252	0.000^**^
LDL-C	Total	237	−0.335 ± 0.417	0.000^**^
	Male	109	−0.335 ± 0.417	0.001^**^
	Female	128	−0.317 ± 0.409	0.012^*^
TG	Total	237	−0.110 ± 0.382	0.000^**^
	Male	109	−0.110 ± 0.382	0.002^**^
	Female	128	−0.114 ± 0.362	0.002^**^

**Notes.**

* *P* < 0.05.

** *P* < 0.01.

TC total cholesterol HDL-C high-density lipoprotein cholesterol LDL-C low-density lipoprotein cholesterol TG triglycerides

### Association between *ADCY3* gene polymorphisms and the effect of HIIT on blood lipid profiles

A linear regression model was developed incorporating gender, age, and baseline blood lipid values as covariates. Utilizing the ADD model, it was determined that the rs2241759 locus exhibited a significant association with the effect of HIIT on LDL-C (*P* = 0.04363) (Table 6). Specifically, volunteers with AG/GG genotypes demonstrated a greater change in LDL-C compared to those with the AA genotype. Gender-specific analysis revealed that male individuals possessing AG/GG genotypes experienced a more substantial alteration in LDL-C than their counterparts with the AA genotype (β = −0.050, *P* = 0.865); conversely, female volunteers with AA/AG genotypes displayed a more pronounced reduction in LDL-C when compared to those carrying the GG genotype (β = −0.050, *P* = 0.194). Furthermore, male volunteers harboring the A allele showed significantly greater variations in TC values relative to carriers of G allele (β = 0.044, *P* = 0.038).

**Table 6:** The polymorphisms of *ADCY3* gene rs2241759 and the effect of HIIT on blood lipid profiles.

Blood lipids	Variable	*n*	AA	AG	GG	Major allele	TEST	*β*_1_	*P*_ 1_	*β*_2_	*P*_ 2_
TC	Total	237	0.108 ± 0.535	0.107 ± 0.535	0.093 ± 0.495	G	ADD	−0.050	0.462	−0.037	0.08073
	Male	109	0.108 ± 0.540	0.106 ± 0.533	0.093 ± 0.500	G	ADD	0.044	0.038^*^		
	Female	128	0.113 ± 0.513	0.113 ± 0.513	0.076 ± 0.493	G	ADD	−0.051	0.146		
HDL-C	Total	237	0.276 ± 0.251	0.275 ± 0.252	0.258 ± 0.242	G	ADD	0.024	0.359	0.0135	0.5086
	Male	109	0.280 ± 0.250	0.277 ± 0.251	0.260 ± 0.240	G	ADD	−0.016	0.426		
	Female	128	0.260 ± 0.253	0.259 ± 0.254	0.257 ± 0.241	G	ADD	0.008	0.830		
LDL-C	Total	237	−0.335 ± 0.417	−0.337 ± 0.418	−0.337 ± 0.411	G	ADD	−0.046	0.500	−0.065	0.04363^*^
	Male	109	−0.335 ± 0.417	−0.339 ± 0.418	−0.337 ± 0.411	G	ADD	−0.050	0.865		
	Female	128	−0.319 ± 0.409	−0.319 ± 0.410	−0.315 ± 0.448	G	ADD	−0.050	0.194		
TG	Total	237	−0.110 ± 0.382	−0.110 ± 0.384	−0.085 ± 0.363	G	ADD	−0.008	0.313	−0.012	0.938
	Male	109	−0.110 ± 0.382	−0.107 ± 0.385	−0.090 ± 0.363	G	ADD	−0.037	0.388		
	Female	128	−0.116 ± 0.362	−0.114 ± 0.363	−0.084 ± 0.366	G	ADD	−0.021	0.189		

**Notes.**

* *P* < 0.05.

** *P* < 0.01.

TC total cholesterol HDL-C high-density lipoprotein cholesterol LDL-C low-density lipoprotein cholesterol TG triglycerides

(*β*_1_/*P*_1_) reflects the outcomes obtained without controlling for the confounding effect of age, gender, and baseline blood lipid profiles. Conversely, (*β*_2_/*P*_2_) represents the results derived from a model that accounts for these phenotypic variables by incorporating age, gender, and baseline blood lipid profiles as covariates.

## Discussion

### Association between *ADCY3* gene polymorphisms and baseline blood lipid profiles

This study found that, prior to training, the TC, LDL-C, and TG in volunteers with AA/AG genotypes at the rs2241759 locus were higher than those observed in volunteers with GG genotypes; however, these differences did not reach statistical significance. Additionally, the HDL-C level in volunteers with GG genotype was significantly greater than that in individuals with AA/AG genotypes. Further analysis by gender indicated that male volunteers carrying the GG genotype exhibited a significantly higher HDL-C value compared to their counterparts with AA/AG genotypes. These findings suggest a potential association between the rs2241759 locus of the *ADCY3* gene and blood lipid profiles.

Obesity is the primary contributor to CAD, with dyslipidemia being a significant characteristic of this condition (Grover et al., 2000). Numerous studies have demonstrated a close association between the *ADCY3* gene and obesity, indicating that an increase in DNA methylation levels of the *ADCY3* gene may trigger the pathogenesis of obesity (Rogne & Taskén, 2014; Wu et al., 2016). This relationship suggests that the *ADCY3* gene is linked to lipid metabolism. Research investigating the correlation between polymorphisms in the *ADCY3* gene and childhood obesity has revealed that rs10187348 and rs4665273 are significantly associated with triglyceride levels (Zhang et al., 2023). These findings align closely with those presented in this article that the polymorphism of the *ADCY3* gene in males has been found to be associated with levels of HDL-C. Furthermore, another study identified a significant association between the GG genotype at the rs6751537 locus within the *ADCY3* gene and CAD, revealing that individuals presenting abnormal blood lipid profiles or high genetic risk at the rs6751527 locus had a CAD prevalence rate 7.43 times higher than those with normal blood lipids or low genetic risk (Ye et al., 2022).

### The effect of HIIT on ameliorating blood lipid profiles

After 12-week of HIIT, the blood lipid profiles of the volunteers demonstrated significant improvement, with TC and HDL-C values significantly increased (*P* < 0.01), while total LDL-C and TG values showed significant decreases (*P* <  0.01). A meta-analysis involving 468 adolescents indicated that, when compared to MICT, HIIT led to a notable increase in HDL-C among adolescents (*P* < 0.05) and a reduction in TC, LDL-C, and TG (Wang et al., 2024). Furthermore, a previous study comparing the effects of HIIT and MICT on blood lipid concentrations in non-diabetic obese young individuals found that HIIT significantly decreased LDL concentrations (−6.23 mg/dL, *P* = 0.05) and TC levels (−7.85 mg/dL, *P* = 0.02), without having a significant effect on HDL or TG concentrations (Mc, Mamikunian & Thorp, 2023). These findings collectively suggest that HIIT can effectively enhance blood lipid status.

Exercise has been shown to enhance insulin sensitivity, mitigate inflammatory responses, and improve cardiovascular function (Flockhart et al., 2023). Inflammatory mediators, such as pro-inflammatory cytokines (TNF-*α*, IL-1β, and IL-8) as well as anti-inflammatory cytokines (IL-10 and TGF-β), can induce insulin resistance without affecting lipid breakdown. Conversely, IL-6 has the capacity to increase insulin sensitivity while promoting lipolysis and fatty acid oxidation (Lang Lehrskov et al., 2018; Petersen & Pedersen, 2005; Plomgaard et al., 2008). A study involving male adolescents demonstrated that after 6-week of short-distance interval training (SIT), there was a significant reduction in inflammatory cytokines including TNF-*α*, IL-1β, and IL-10 alongside an increase in IL-6 levels (Wang et al., 2023). Numerous studies suggest that the health benefits of exercise may be attributable to its ability to lower levels of inflammatory cytokines. For instance, elderly individuals with CAD or those at high risk for CAD may exhibit decreased pro-inflammatory cytokine levels along with elevated IL-6 following physical activity (De Farias et al., 2021; Ho et al., 2013; Pedersen, 2017). Moreover, when compared with healthy control subjects, patients with T2DM who engaged in low-volume high-intensity interval training (LVHIIT) exhibited a significant reduction in insulin resistance levels (RR = −1.34; 95% CI [−2.59–0.10]; *P* = 0.03), ultimately leading to improved blood lipid profiles (Peng et al., 2022). Resistance training also resulted in notable decreases in TG, TC, and LDL-C among T2DM patients—an outcome associated with a marked reduction in the homeostasis model assessment (HOMA) of insulin resistance index scores (Fan, Lin & Kim, 2023). Therefore, engaging in 12-week of HIIT could lead to improvements in blood lipid status through reductions in inflammatory cytokine concentrations while simultaneously enhancing insulin sensitivity among volunteers—facilitating fat breakdown and promoting fatty acid oxidation.

In this study, the TC value following HIIT training was found to be higher than that measured prior to the training. This finding contradicts some of the results presented in earlier literature (Mc, Mamikunian & Thorp, 2023; Wang & Ye, 2024). Nonetheless, other studies have indicated that after 6-week of moderate to high-intensity aerobic sprint training, there is an increase in the TC values among young males and females aged 20–28; however, these changes were not statistically significant (*P* > 0.05) (Wong et al., 2024). The potential reasons for these discrepancies may include: (1) Research indicates that short-term HIIT does not significantly improve volunteers’ fat loss outcomes without the enforcement of strict dietary restrictions (Xu et al., 2022). (2) HIIT stimulates the production of cholesterol in both the liver and intestinal mucosa, facilitating the synthesis of steroid hormones such as adrenal cortex hormone, testosterone, and progesterone. This process enhances insulin sensitivity in skeletal muscle and adipose tissue, thereby improving exercise performance and promoting fat breakdown (Besqueut-Rougerie et al., 2024; Dos Santos et al., 2024; Su et al., 2019). Furthermore, steroid hormones increase glycogen consumption in muscles during exercise, which promotes continuous fat catabolism to synthesize glycogen during the recovery phase (Hu et al., 2022; Smith, Hopkins & Lowe, 2011). HIIT is characterized by elevated excess post-exercise oxygen consumption (EPOC) and sustained high levels of steroid hormones for a period following exercise. These factors ultimately accelerate fat breakdown and enhance energy expenditure (Lame-Jouybari & Abbasalizad-Farhangi, 2024; Wang & Ye, 2024). Consequently, after HIIT sessions, both the liver and intestinal mucosa may continue to produce cholesterol for further synthesis of steroid hormones, resulting in an increase in serum TC levels. (3) A recent mouse study has demonstrated that HIIT significantly enhances the transcription and translation levels of peroxisome proliferator-activated receptors (PPARs), which are critical signaling molecules involved in regulating fatty acid and cholesterol synthesis (Gu et al., 2022). Additionally, another investigation indicated that HIIT increases PPAR transcription levels by a striking factor of 13 times (Trewin et al., 2018). Acetyl CoA serves as a fundamental substrate for TC synthesis. The primary route for synthesizing acetyl CoA is through glucose oxidation, which consists of glycolysis breaking down glucose into pyruvate. This pyruvate subsequently enters the mitochondria to undergo oxidative decarboxylation, yielding acetyl CoA. This pathway is also pivotal for ATP production within the body (Xie et al., 2024). Following HIIT sessions, there is continuous oxidation of glucose observed (Flockhart et al., 2023). Moreover, triglycerides can partially facilitate the repair of muscle cell damage incurred from HIIT activities (Yamauchi et al., 2023). Consequently, it is hypothesized that following HIIT, a substantial amount of pyruvate derived from decomposed glucose may enter the mitochondria to be converted into acetyl CoA, leading to enhanced TC synthesis for repairing damaged muscle cells. It should also be highlighted that TC plays an essential role in the physical development of adolescents; furthermore, the normal range for TC values in healthy adults span from 3.00 to 5.70 mmol/L, indicating this variability remains within acceptable limits.

### Association between *ADCY3* gene polymorphisms and the effect of HIIT on blood lipid profiles

**Figure 2:**
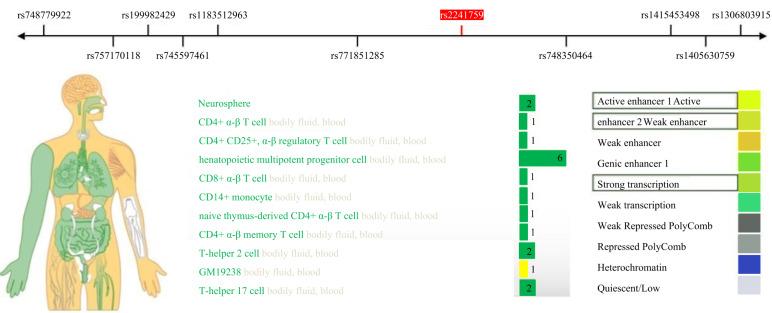
Expression activity of the ADCY3 gene rs2241759 locus in human tissues.

This study identified a significant correlation between the rs2241759 locus and the training effects on LDL-C. Specifically, individuals with AG/GG genotypes exhibited greater variability in LDL-C than those with the AA genotype. These findings suggest that the rs2241759 locus of the *ADCY3* gene is associated with sensitivity to lipid changes following HIIT. The effects of HIIT on blood lipids demonstrate considerable variability across different races, as well as among individuals within the same race and gender; this variation is predominantly influenced by genetic factors (Bouchard et al., 2015; Di Gioia et al., 2024). By querying the RegulomeDB (http://www.regulomedb.org/) database, we identified that the rs2241759 locus of the *ADCY3* gene exhibits significant transcriptional and enhancer activity in immune cells, bodily fluids, and blood (Fig. 2). It is hypothesized that immune cells play a pivotal role in mediating the effects of rs2241759 and HIIT on blood lipids. HIIT has been shown to enhance both the activity and phagocytic capacity of cytotoxic immune cells, specifically CD8+ T cells (Barreto et al., 2024). Furthermore, genome-wide association studies (GWAS) revealed that the rs6713978 and rs13410999 SNPs within the *ADCY3* gene are expressed in adipose tissue and blood; their expression levels in these tissues correlate with BMI. Additionally, these two SNPs influence BMI through mechanisms involving immune proteins associated with natural killer cell-mediated cytotoxicity (NK cells) (Wu et al., 2022). The cytotoxic immune mechanism induced by Sigma-2 receptors increases free cholesterol content within lysosomes (Takchi et al., 2024). These findings suggest that the *ADCY3* gene rs2241759 locus may exert an effect on blood lipid profiles *via* mechanisms involving immune-mediated cytotoxicity, which related to HIIT.

Gender analysis showed that among men, the change value of LDL-C in individuals with AG/GG genotypes was higher than that in those with AA genotypes; compared with GG individuals, female individuals with AA/AG genotypes had a greater decrease in LDL-C. However, there was no statistical difference. The glucokinase regulatory protein (GCKR) rs1260326 polymorphism is related to metabolic characteristics. Compared with the C/C genotype, male individuals with the T/T genotype had lower body weight, body mass index (BMI) and skeletal mass index (Sakamoto et al., 2024). The fatty-acid amide hydrolase (FAAH) rs324420 A allele was significantly associated with improved exercise performance (Silva et al., 2022). The research results and previous studies indicate that gene polymorphisms can affect the exercise response of sexual dimorphism. The ADCY3-cAMP-PKA signaling pathway plays a crucial role in lipolysis (Liang et al., 2016). PKA can phosphorylate hormone-sensitive lipase (HSL) and activate it, thereby initiating the lipolysis process and decomposing triglycerides into glycerol and free fatty acids (Yu et al., 2024). In male adipocytes, the presence of androgens may enhance the sensitivity of ADCY3 to certain signals, making ADCY3 more easily activated and thus promoting lipolysis (Khani et al., 2024). Androgens can increase ADCY3 activity by regulating the expression of the ADCY3 gene or its interaction with G proteins, thereby accelerating the breakdown of abdominal fat (Johansson et al., 2013). In female adipocytes, estrogen may have different effects on the ADCY3-cAMP-PKA signaling pathway (Zheng et al., 2023). Estrogen may regulate the ADCY3 activity or substrate specificity of PKA by binding to estrogen receptors (Zhang & Liu, 2015; Zheng et al., 2024). For example, estrogen may inhibit ADCY3 activity, making it relatively difficult to initiate lipolysis in female adipocytes, especially in gluteal adipocytes, which may be one of the reasons why it is more difficult for women to reduce gluteal fat through exercise (Anstead, Wilson & Katzenellenbogen, 1989; Chen et al., 2020; Mandrup et al., 2020).

This study aims to investigate the association between polymorphisms in the *ADCY3* gene and the effects of HIIT on blood lipid profiles among Chinese Han youth. The ultimate goal is to elucidate the molecular mechanisms through which exercise promotes health, thereby providing a theoretical foundation for developing personalized exercise training programs aimed at preventing and treating CAD. However, this study has certain limitations: (1) We were unable to strictly control the dietary habits of the subjects. It is well established that dietary composition and appetite can significantly influence the outcomes of exercise training; thus, future research should focus on enhancing dietary control. (2) The sample size in this current study was relatively small for young individuals in China; therefore, we plan to include a larger volunteers pool in subsequent studies.

## Conclusion

The 12-week HIIT program effectively improved the blood lipid profiles of young college students, significantly increasing TC and HDL-C levels, while simultaneously decreasing LDL-C and TG levels. Additionally, male volunteers who carried the rs2241759 locus of the *ADCY3* gene exhibited a correlation with their baseline HDL-C values; those with the AA or AG genotype had lower HDL-C values compared to individuals with the GG genotype. Moreover, the rs2241759 locus of the *ADCY3* gene is associated with sensitivity to HIIT concerning blood lipid alterations.

##  Supplemental Information

10.7717/peerj.19271/supp-1 Supplemental Information 1 Data

10.7717/peerj.19271/supp-2 Supplemental Information 2 ADCY3 gene information

10.7717/peerj.19271/supp-3 Supplemental Information 3 Health survey and physical activity questionnaire (English)

10.7717/peerj.19271/supp-4 Supplemental Information 4 Health survey and physical activity questionnaire (original)

## Additional Information and Declarations

### Data Availability

The following information was supplied regarding data availability: The raw measurements are available in the Supplemental Files.
