# Supplementary material for: Association between polymorphisms of the adenylate cyclase 3 gene rs2241759 and the effect of high-intensity interval training on blood lipid profiles
Source: PeerJ. 2025 Apr 11;13:e19271. doi: 10.7717/peerj.19271 (PMC11995890; doi:10.7717/peerj.19271)
Supplement: Supplemental Information 3 [file peerj-13-19271-s003.pdf]

3 Sports Risk Screening Questionnaire Survey

Health and Physical Activity Survey Questionnaire

Hello, this is a survey questionnaire about "Health and Physical Activity" aimed at understanding your health, lifestyle habits, and physical exercise status. Please fill in the form truthfully based on your own situation. The final result will only be used for academic research and will not disclose your personal information. Thank you for your cooperation!

Please select the answer number and fill in the corresponding "□" or fill in the relevant content in the "　　".

1. Ethnicity: □

① Han Chinese　　② Other (please fill in)__________

2. Your occupation (for retired individuals, fill in the occupation before retirement): □

① Leaders of state organs, party mass organizations, enterprises (including private enterprises), and public institutions

② Professional technical personnel

③ Office staff and related personnel

④ Business and service industry personnel

⑤ Production personnel in agriculture, forestry, animal husbandry, fishery and water conservancy industries

⑥ Production and transportation equipment operators and related personnel ⑦ Military personnel

⑧ Other3.

Education □

① Primary school and below ② Junior high school ③ High school or vocational school ④ University (including junior college) ⑤ Master's degree or above

4. Household per capita monthly income (after tax income, RMB: ￥,Yuan) □

①1000 或以下　②1001-2000　③2001-3000　④3001-4000

动健康促进效果个体差异的生物学机制与健身指导方

⑤4001-5000　⑥5001-8000　⑦8001-10000　⑧10000

动健康促进效果个体差异的生物学机制与健身指导方

How is your sleep condition? □

① Poor ② average ③ good

6.Your usual daily sleep (including nap time) is:_______hour_____min

7.Do you smoke? □

① Yes ② has quit smoking ③ No, if the answer is "no", please skip to question 9

8.Your smoking situation: (Only fill in 1 item)

① I have quit smoking; Stop smoking for the Chinese New Year, previously smoking for the Chinese New Year

② Smoking now, but not every day; Smoking for about years, now the average smoking volume is cigarettes per week

③ Smoking every day now; Smoking for about years, the current average smoking volume is cigarettes per day

9. Do you drink alcohol? □

① Yes ② No, if the answer is "no", please skip to question 11

10.How much alcohol have you consumed on average each time in the past two weeks?

(Can be filled in more)

① Beer bottles, milliliters per bottle

② Red wine or two types of wine

③ Two spirits (Baijiu, foreign wine, etc.)

11.How did you feel about your physical condition in the month before filling out the form? □

① Very good ② Good ③ Fair ④ Poor ⑤ Very poor

12.Did you feel any pressure in the month before filling out the form? □

① More than 15 days ② 7-15 days ③ 1-6 days ④ none ⑤ unclear

运动健康促进效果个体差异的生物学机制与健身指导方

13.Does your immediate relative have cardiovascular disease? □

① None

② Yes, the age of onset for direct relatives is under 60 years old

③ Yes, the age of onset for direct relatives is over 60 years old

14.Have you self tested or been informed by medical staff that you have hypertension? □

①Yes ②No

15.Have you ever been informed by medical staff that you have blood lipid abnormalities (hyperlipidemia)? □

①Yes ②No

16.Diseases that have been previously or currently diagnosed by a doctor (multiple choice)

① Heart disease ② Peripheral vascular disease ③ Cerebrovascular disease

④ Hypotension ⑤ Rheumatic fever ⑥ Liver disease (excluding fatty liver)

⑦ Type I or II diabetes ⑧ chronic obstructive emphysema ⑨ interstitial lung disease or cystic fibrosis
⑩ Thyroid dysfunction 1 Kidney disease 2 Asthma ⑬not at all

17. Do you have the following signs or symptoms (multiple choice).
① Palpitations or tachycardia ② Orthostatic breathing or paroxysmal nocturnal dyspnea ③ Obvious heart murmurs
④ Abnormal fatigue or shortness of breath during quiet state or mild vigorous activity ⑤ Ankle injury or swelling
⑥ Frequent chest discomfort ⑦ Dizziness, dizziness or fainting (especially during exercise)
⑧ There have been no signs or symptoms mentioned above

18.Have you engaged in regular sports and fitness activities in the past year? □

① No (for those who choose "no", skip directly to question 22)

② Yes

运动健康促进效果个体差异的生物学机制与健身指导方

19.Fill in your fitness status based on the intensity of your exercise and whether you engage in strength training (multiple choices are allowed)

19-1	Is it a high-intensity endurance exercise? (Rapid breathing and significantly increased heart rate during exercise)<br>Quick, sweating a lot)<br>① Yes... If you choose this option, continue answering question ② in the right column. No	Exercise Program	Frequency/ month	Every time (minutes)	Persist for a certain period of time (Year)
19-2	Is it a moderate intensity endurance exercise? (Breathing and heartbeat increase during exercise, slight sweating)<br>① Yes... If you choose this option, continue answering the questions in the right column<br>② No	Exercise Program	Frequency/ month	Every time (minutes)	Persist for a certain period of time (Year)
19-3	Have you engaged in low-intensity endurance exercises? (There is little change in breathing and heartbeat during exercise compared to when not exercising)<br>①Yes If you choose this option, continue answering the questions in the right column<br>②No	Exercise Program	Frequency/ month	Every time (minutes)	Persist for a certain period of time (Year)
19-4	Have you engaged in strength training? (Strength exercises for muscles in the upper and lower limbs, chest, back, neck, and other areas) □<br>①Yes If you choose this option, continue answering the questions in the right column<br>②No	Exercise Program	Frequency/ month	Every time (minutes)	Persist for a certain period of time (Year)

运动健康促进效果个体差异的生物学机制与健身指导方

20.Have you ever noticed changes in your heart rate during exercise? □

①Yes ②No

21.Have you ever considered seeking professional guidance on your exercise and fitness?

□ ①Yes ②No

运动健康促进效果个体差异的生物学机制与健身指导方

22.What is the reason why you don't participate in sports and fitness? (If you have already participated, do not answer this question. This question can be multiple-choice)

① Not liked ② no time ③ no venue or equipment ④ unsuitable physical condition ⑤ insufficient economic ability

⑥ Lack of technical guidance ⑦ Lack of sports companions ⑧ Afraid of being laughed ⑨ Other

23. Static Activity Behavior Survey

What is your average daily TV viewing time in hours and minutes

What is your average daily computer usage time in hours and minutes

What is your average daily time spent reading (paper books) in hours and minutes

What is your average daily time playing video games (excluding computer games): hours minutes

Other sitting based activities (excluding sleep and eating) such as: hours and minutes

24.(This question is limited to females) Has amenorrhea occurred □

①No

②The age of amenorrhea is year old

运动健康促进效果个体差异的生物学机制与健身指导方

In order to have a more detailed understanding of your health condition, please patiently fill out the following form. If you have any questions, please communicate with the researchers!

25. Have you been informed by a doctor that you have the following diseases? If so, please provide information such as the time of illness, family history, and laboratory test results.

	Do you have this disease<br>1. Yes<br>2. No	Duration of illness: XXXX years	What measures have you taken to treat the disease? (Multiple Choice)<br>1. Medication<br>2. Control diet<br>3. Exercise<br>4. Surgery<br>5. Other	Have your parents ever suffered from this disease?<br>1. Yes, onset under the age of 60<br>2. Yes, the disease occurs when the patient is over 60 years old<br>3. No	Recent laboratory and testing results
diabetes					Fasting blood glucose： mmol/L
hypertension					systolic pressure： mmHg<br>diastolic pressure： mmHg
dyslipidemia					TC： mmol/L<br>TG： mmol/L<br>LDL: mmol/L<br>HDL: mmol/L
fatty liver					① Mild ② Moderate ③ Severe：□
osteoporosis					
coronary heart disease					
Stroke					
osteoarthritis					
anaemia					hemoglobin： g/L

运动健康促进效果个体差异的生物学机制与健身指导

26. Survey on past physical exercise habits. Please recall if you have engaged in regular physical exercise since the age of 20? What projects have you practiced before, frequency and time of practice.

If you have no previous exercise experience, please indicate "none" in the exercise program column. If you have already filled in the sports exercise information in question 19, do not repeat it here.
Example of filling out the form: If you played volleyball at the age of 20 and swam between the ages of 40-45, fill in "volleyball" and "swimming" in the "exercise program" column, and fill in the corresponding number of times, time, and years of practice in the tables for these two age groups. See the following table for examples.

Exercise Program	20-29 years		
	How many months/year	How many times/year	Time per session (minutes)
Volleyball (Example)	12	8	
Swimming (for example)			
Remarks: (Provide an explanation for the			

动健康促进效果个体差异生物学机制与健身指导方案

above filled in content)	

动健康促进效果个体差异的生物学机制与健身指导方案

4.Statistical Table of Health and Exercise Risk Assessment

Statistical Table of Health and Exercise Risk Assessment

<table>
<tr><td colspan="2">Name Number</td><td></td><td></td><td></td></tr>
<tr><td colspan="2"></td><td>No</td><td>Yes</td><td></td></tr>
<tr><td colspan="2">Diseases currently or previously reported by doctors (Question 16)</td><td>□</td><td>□</td><td>If there is, it will not be used as a research subject</td></tr>
<tr><td colspan="2">Signs or symptoms of existence (Question 17)</td><td>□</td><td>□</td><td>If there is, it will not be used as a research subject</td></tr>
<tr><td rowspan="7">Risk factors</td><td>Direct relatives with cardiovascular disease (Question 13)</td><td>□</td><td>□</td><td>for your reference</td></tr>
<tr><td>Is the blood pressure ≥ 160/100mmHg (Question 14, and refer to the physical fitness test, quiet blood pressure test)</td><td>□</td><td>□</td><td>If there is, it will not be used as a research subject</td></tr>
<tr><td>Is there any abnormality in blood lipids? (Question 15)*</td><td>□</td><td>□</td><td rowspan="5">As a reference for sports risk assessment</td></tr>
<tr><td>Are you currently smoking or have you quit smoking for less than 6 months? (Question 8)</td><td>□</td><td>□</td></tr>
<tr><td>Lack of regular physical exercise habits (Question 18)</td><td>□</td><td>□</td></tr>
<tr><td>Whether obese (BMI ≥ $28kg/m^2$)</td><td>□</td><td>□</td></tr>
<tr><td>Male ≥ 45 years old or female ≥ 55 years old</td><td>□</td><td>□</td></tr>
<tr><td colspan="5">The more risk factors one has, the greater the risk of participating in GXT testing may be; For individuals with high risk factors, more attention should be paid to changes in the subjects' reactions during high-intensity exercise testing* If HDL-C>60mg/dl or 1.55mmol/L, the risk assessment of exercise testing can be reduced by one level.</td></tr>
</table>

Please carefully fill in the questionnaire based on the survey results and blood test results!

5 GPAQ Physical Activity Questionnaire

Core content: Physical activity			
Next, I would like to inquire about the amount of time you typically spend on various physical activities each week. Please answer the following questions (even if you think you don't engage in physical activity regularly).<br>Firstly, let's talk about physical activity at work. Work refers to the paid or unpaid work that you must complete, including studying and training, household chores, harvesting food/grains, fishing or hunting for food, and finding a job. Add other example sentences as needed<br>Regarding vigorous activity, it refers to high load physical activity that causes a significant increase in breathing and heartbeat. Moderate intensity activity refers to physical activity with a certain load that causes a slight increase in breathing and heartbeat.			
**questions**		**answers**	**code**
**Physical activity during work**			
**54**	Does your job require intense activity that causes a significant increase in breathing and heartbeat, such as carrying or lifting heavy objects, digging or construction work, for at least 10 minutes?<br>[Insert the example knife (using the illustrated card)	Yes 1<br>No, transfer to P4	P1
55	How many days per week do you typically engage in strenuous activities in your work?	days	P2
56	How much intense activity do you usually do at work every day?	hour：min L<br>hour min	P3 (a-b)
57	Does your job require moderate intensity activities that cause a slight increase in breathing and heartbeat, such as brisk walking [carrying lighter items] for at least 10 minutes?	Yes 1<br>No 2 transfer to P7	P4
58	How many days per week do you usually work for moderate intensity activities?	days	P5
59	How much moderate intensity activity do you usually do at work every day?	hour：min L<br>m<br>in	P6 (a-b)
**Physical activity during transportation**			
The following questions do not include physical activity during the aforementioned work.<br>Now I want to inquire about your usual mode of transportation. For example, going to work, shopping, going to the market, etc. [Add other examples as needed			
60	Do you walk or ride a bike for at least 10 minutes when you go somewhere?	Yes 1<br>No2 transfer to P10	P7
61	How many days per week do you usually walk or bike from one location to another for at least 10 minutes?	days	P8
62	How much time do you usually spend walking or cycling in terms of transportation every day?	hour：min	P9 (a-b)
**Recreational physical activity**			
The following questions do not include physical activities during the aforementioned work and transportation processes.<br>Now I am asking you questions about sports, fitness, and recreational physical activities (leisure) [insert relevant examples]			
63	You engage in vigorous exercise that significantly increases your breathing and heartbeat<br>Do fitness and recreational (leisure) physical activities last for at least 10 minutes?<br>[insert example] (using illustrated cards)?	Yes 1<br>No 2 transfer to P13	P10
64	How many days per week do you typically engage in vigorous exercise, fitness, and recreational physical activities?	days	P11
65	How much time do you usually spend on vigorous exercise, fitness, and recreational physical activities every day?	Hou：min :1	P12 (a-b)

**66**	You engage in moderate intensity exercise that causes a slight increase in your breathing and heartbeat<br>Do physical activities such as brisk walking (cycling, swimming, volleyball) last for at least 10 minutes or more, including exercise, fitness, and recreational physical activities (leisure)?<br>[Insert Example] (Using Illustrative Cards)	Yes 1<br>No 2 transfer to P16	P13
**67**	How many days per week do you typically engage in moderate intensity exercise, fitness, and recreational physical activities?	days	P14
**68**	How much time do you usually spend on moderate intensity exercise, fitness, and recreational (leisure) physical activities every day?	Hour:min	P15 (a-b)

**Extended content: Physical activity**				
**Sedentary habit**				
The following questions are about the time spent sitting or leaning against each other while working, at home, in transportation, or meeting friends. This includes sitting at a desk, sitting with friends, taking a car, bus, train, reading, playing poker, or watching TV, but does not include sleeping time.<br>(Insert Example) (Using Illustrative Cards)				
69	How much time do you usually spend sitting or leaning against each other every day?	Hour:min		P16 (a-b)

WH

6 Physical Activity Hazard Checklist

(This form is used for self-assessment of physical condition before each exercise)

Physical Activity Hazard Checklist

	project	answers	
1	Do you have any heart problems	Yes	No
2	Is exercise suffocating and chest tightness?	Yes	No
3	Do you experience chest pain or irregular heartbeat when not exercising?	Yes	No
4	Do you experience dizziness or loss of consciousness?	Yes	No
5	Do you have a family history of sudden death for unknown reasons?	Yes	No
6	Do you have any movement disorders?	Yes	No
7	Will lower back pain worsen during exercise?	Yes	No

Even if one of the answers is' yes', the risk increases during exercise and should be consulted with a doctor.
If all answers are 'no', conduct a 'pre exercise self risk screening'

Self examination checklist before starting exercise

	project	answers	
1	Strong pain in the feet and waist	Yes	No
2	Fever	Yes	No
3	Weakness	Yes	No
4	Gross	Yes	No
5	Headache	Yes	No
6	Tinnitus	Yes	No
7	Tired	Yes	No
8	Insomnia	Yes	No
9	No appetite	Yes	No
10	Drinking alcohol for two consecutive days and feeling unwell	Yes	No
11	Gastrointestinal discomfort	Yes	No
12	be short of breath	Yes	No
13	Cold	Yes	No
14	Chest pain	Yes	No
15	signs of heatstroke	Yes	No
