# Supplementary material for: Association between polymorphisms of the adenylate cyclase 3 gene rs2241759 and the effect of high-intensity interval training on blood lipid profiles
Source: PeerJ. 2025 Apr 11;13:e19271. doi: 10.7717/peerj.19271 (PMC11995890; doi:10.7717/peerj.19271)

## Slide 1
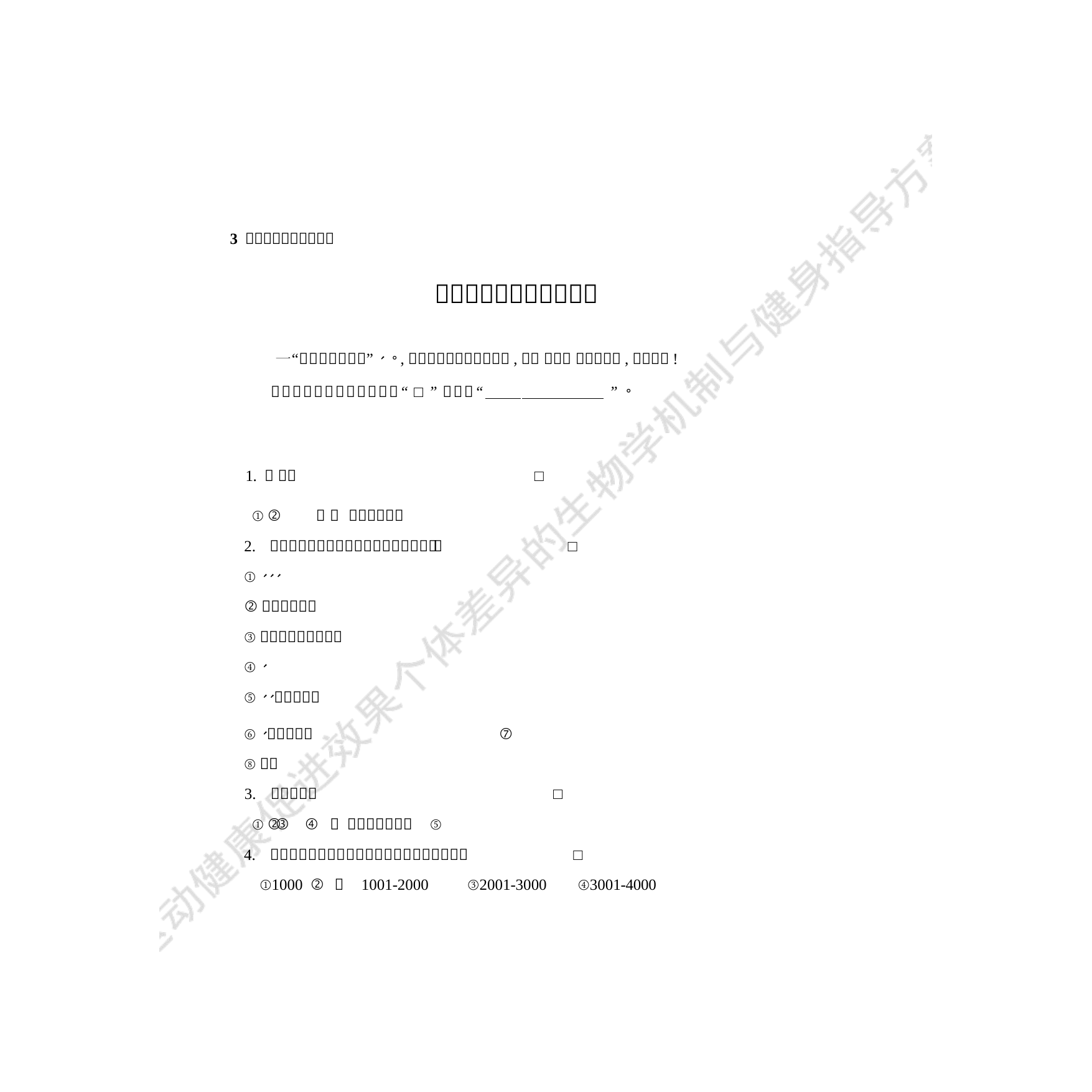

3 运动风险筛查问卷调查
健康与身体活动调查问卷
您好，这是一项关于“健康与身体活动” 的调查问卷，旨在了解您的健康、生活习惯以 及体育锻炼状况。请根据您的自身情况如实填写,最终结果仅作为学术研究,不会 外泄您 的个人资料,谢谢合作!
请选择答案序号填入相应的“ □ ” 内或在“ ” 中填写相关内容。
1. 民 族： □
①汉族 ②其他（请填写）
2. 您的职业（已退休者，填退休前的职业）： □
①国家机关、党群组织、企业（含私营企业）、事业单位负责人
②专业技术人员
③办事人员和有关人员
④商业、服务业人员
⑤农、林、牧渔水利业生产人员
⑥生产、运输设备操作人员及有关人员 ⑦军人
⑧其他
3. 受教育程度 □
①小学及以下 ②初中 ③高中或中专 ④大学（含大专） ⑤硕士及以上
4. 家庭人均月收入情况（税后收入，人民币：元） □
①1000 或以下 ②1001-2000 ③2001-3000 ④3001-4000

## Slide 2
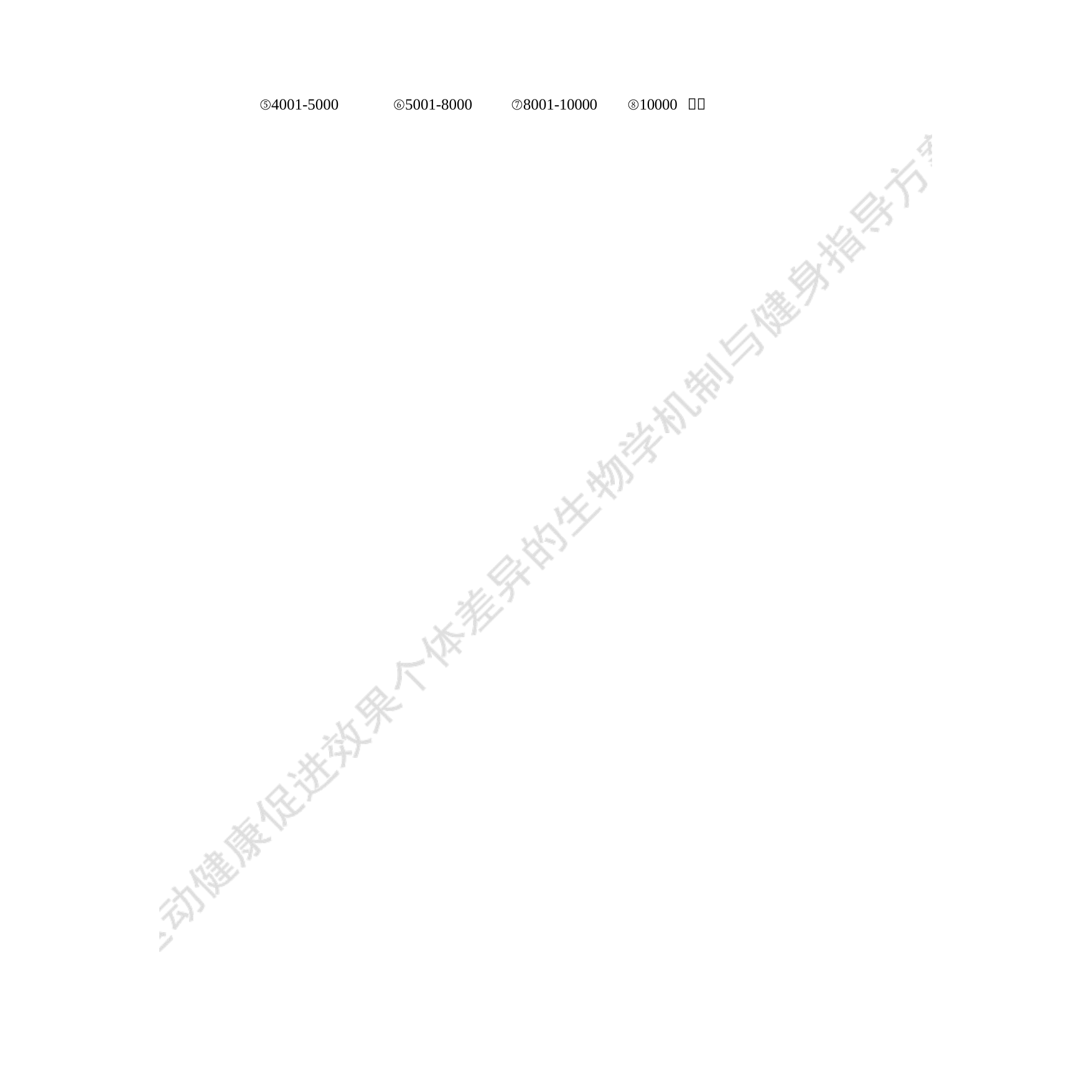

⑤4001-5000 ⑥5001-8000 ⑦8001-10000 ⑧10000 以上

## Slide 3
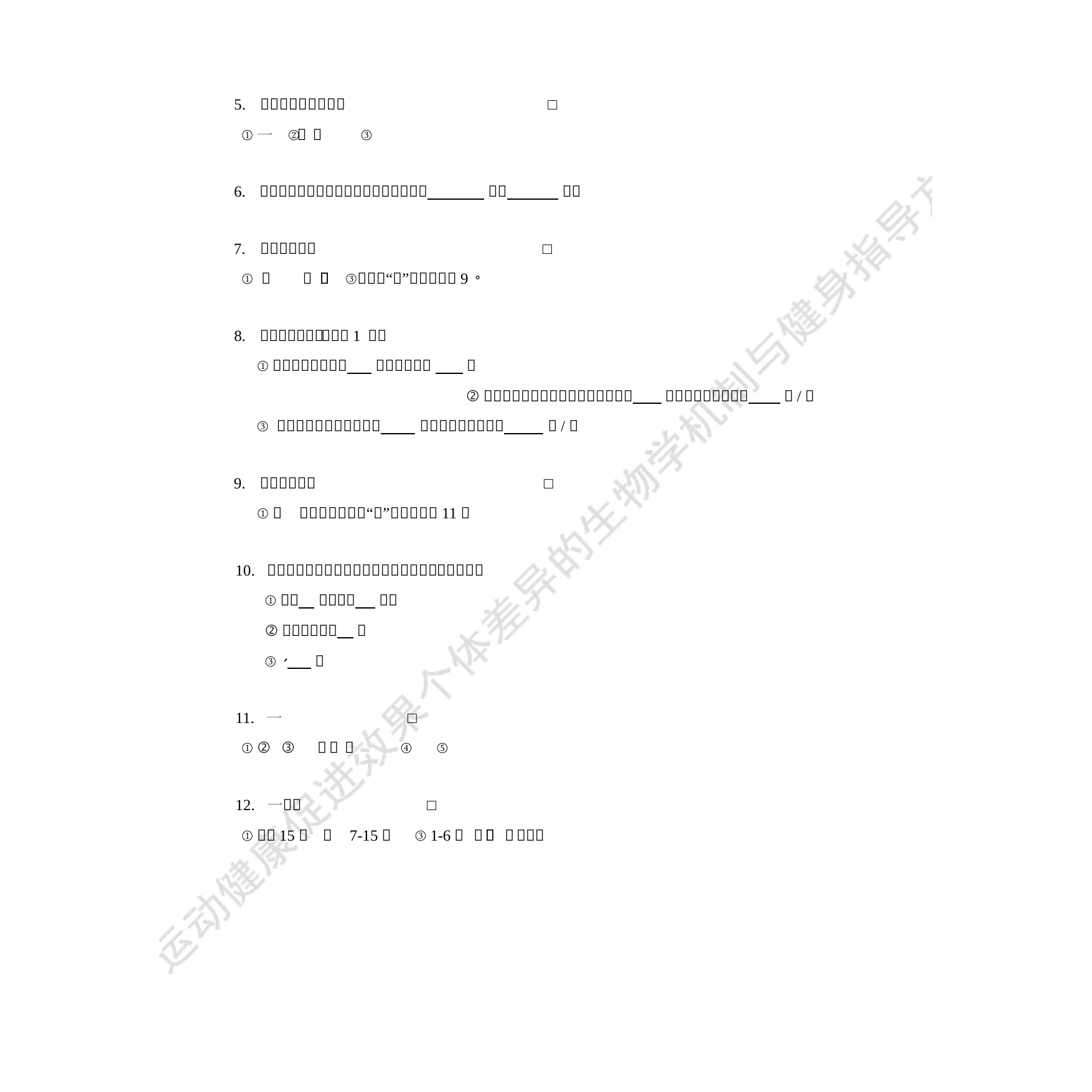

5. 您的睡眠状况如何？ □
①差 ②一般 ③好
6. 您通常每天的睡眠（包括午睡）时间为： 小时 分钟
7. 您是否吸烟？ □
① 是 ②已戒烟 ③否，如果回答“否”，请跳到第9题。
8. 您吸烟的情况：（只填1 项）
①已戒烟；停止吸烟 年，之前吸过 年
②现在吸烟，但不是每天吸；吸烟大约 年，现在平均吸烟量 支/周
③ 现在每天吸烟；吸烟大约 年，现在平均吸烟量 支/天
9. 您是否饮酒？ □
①是 ②否，如果回答“否”，请跳到第11题
10. 过去两个星期，您平均每次喝了多少酒？（可多填）
①啤酒 瓶，每瓶 毫升
②红酒或葡萄酒 两
③烈酒（白酒、洋酒等） 两
11. 填表前一个月内，您感觉身体状况如何？ □
①很好 ②良好 ③一般 ④差 ⑤很差
12. 填表前一个月内，您是否感觉到有压力？ □
①多于15天 ②7-15天 ③1-6天 ④没有 ⑤不清楚

## Slide 4
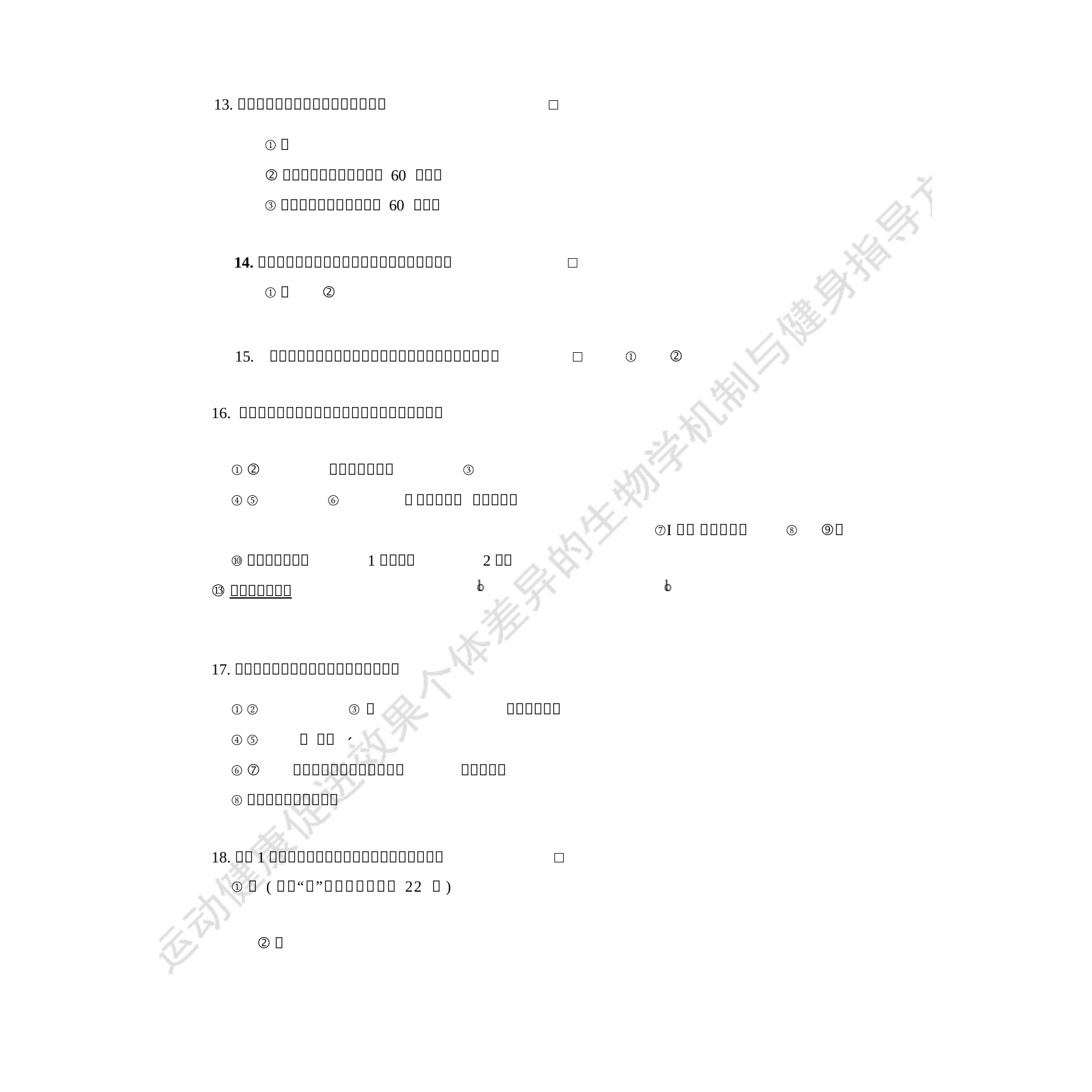

13.您的直系亲属有无心血管系统疾病？ □
①无
②有，直系亲属发病年龄在 60 岁以下
③有，直系亲属发病年龄在 60 岁以上
14.您是否自测或被医务工作人员告知患有高血压？ □
①否 ②是
15. 您是否曾被医务工作人员告知患有血脂异常（高血脂）？ □ ①否 ②是
16. 现在或以前曾经被医生告知患有的疾病（可多选）
①心脏疾病 ②外周血管疾病 ③脑血管疾病
④低血压 ⑤风湿热 ⑥肝病（不包括脂肪肝）
⑦I型或 Ⅱ型糖尿病 ⑧慢性阻塞性肺气肿 ⑨间质性肺病或囊性纤维化
⑩甲状腺功能异常 1肾脏疾病 2哮喘
⑬未患过上述疾病
17.您是否存在以下体征或症状（可多选）。
①心悸或心动过速 ②端坐呼吸或夜间阵发性呼吸困难 ③明显心脏杂音
④安静状态或轻度用力活动时感到异常疲劳或气短 ⑤踝部损伤、肿胀
⑥经常性胸部不适 ⑦头晕眼花或晕厥（特别是运动期间）
⑧未有过上述体征或症状
18.最近1年，您是否从事过规律性的运动健身活动？ □
①否 (选择“否”者，直接跳到第 22 题)
②是

## Slide 5
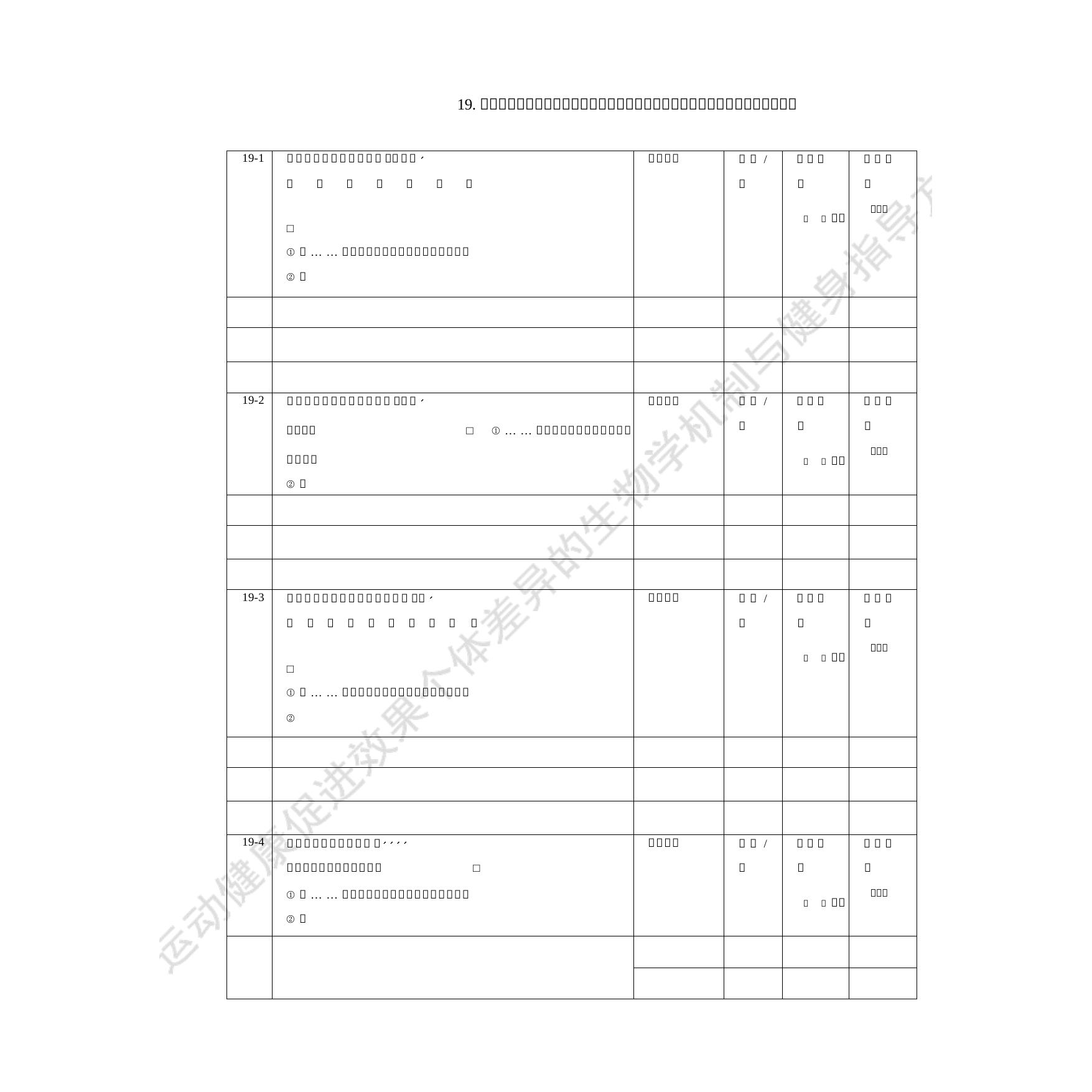

19.按照运动强度的不同以及是否从事力量练习，填写您的运动健身情况（可多选）
| 19-1 | 是大强度的耐力锻炼吗？ （锻炼时呼吸急促、心跳明显加 快 ， 出 汗 较 多 ） □ ①是 … … 如选择此项，继续回答右栏内的问题 ②否 | 锻炼项目 | 次 数 / 月 | 每 次 时 间 （ 分 钟） | 坚 持 年 限 （年） |
| --- | --- | --- | --- | --- | --- |
| | | | | | |
| | | | | | |
| | | | | | |
| 19-2 | 是中等强度的耐力锻炼吗？ （锻炼时呼吸、心跳加快，微 微出汗） □ ①是 … … 如选择此项，继续回答右栏内的问题 ②否 | 锻炼项目 | 次 数 / 月 | 每 次 时 间 （ 分 钟） | 坚 持 年 限 （年） |
| | | | | | |
| | | | | | |
| | | | | | |
| 19-3 | 是否从事过小强度的耐力锻炼？ （锻炼时呼吸、心跳与不 锻 炼 时 比 ， 变 化 不 大 ） □ ①是 … … 如选择此项，继续回答右栏内的问题 ② | 锻炼项目 | 次 数 / 月 | 每 次 时 间 （ 分 钟） | 坚 持 年 限 （年） |
| | | | | | |
| | | | | | |
| | | | | | |
| 19-4 | 是否从事过力量锻炼？ （对上肢、下肢、胸、背部、颈部等 部位的肌肉进行力量锻炼） □ ①是 … … 如选择此项，继续回答右栏内的问题 ②否 | 锻炼内容 | 次 数 / 月 | 每 次 时 间 （ 分 钟） | 坚 持 年 限 （年） |
| | | | | | |
| | | | | | |

## Slide 6
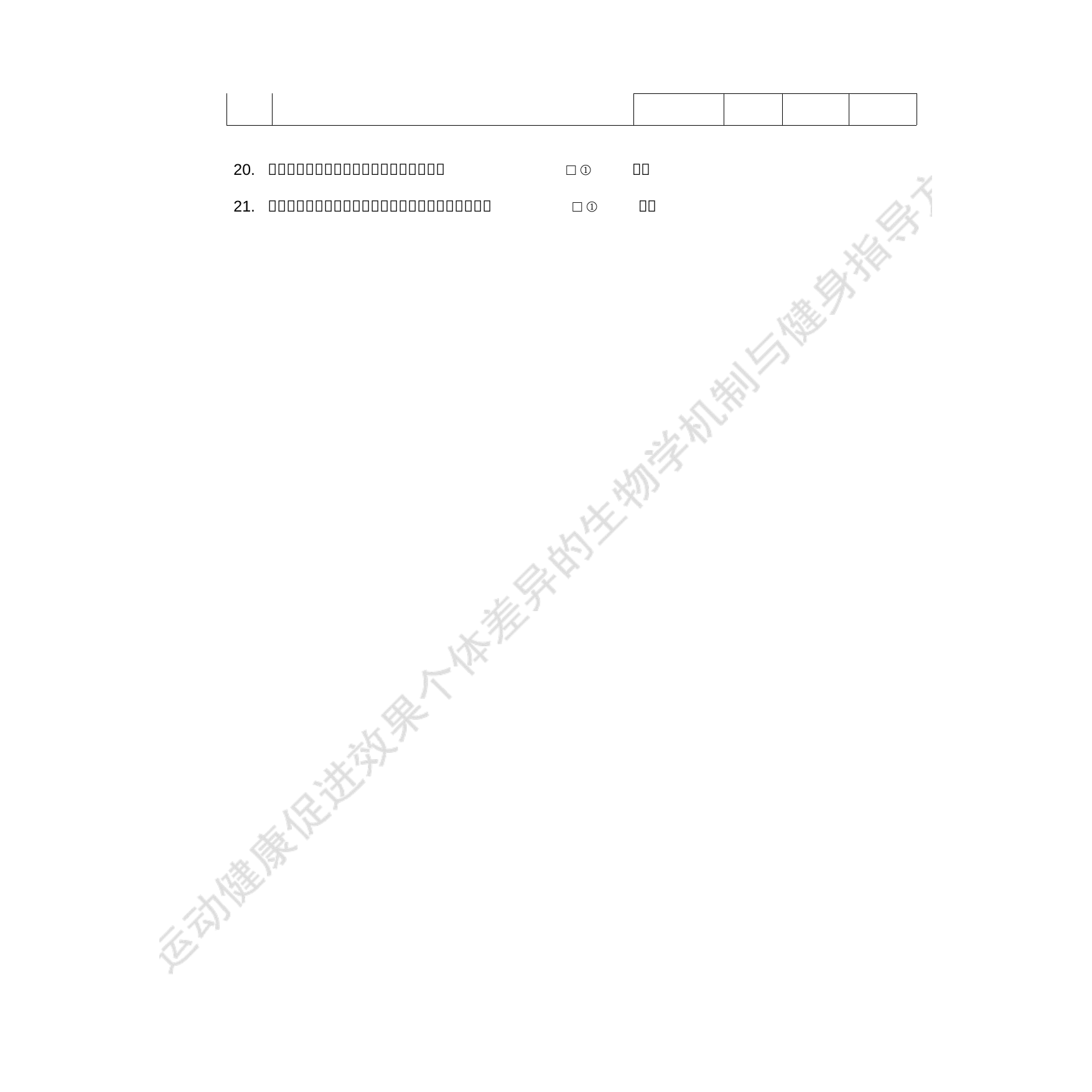

| | | | | | |
| --- | --- | --- | --- | --- | --- |
20. 您在运动过程中是否注意过您的心率变化？ □ ①是 ②否
21. 您是否想过请专业人员对您的运动健身进行科学指导？ □ ①是 ②否

## Slide 7
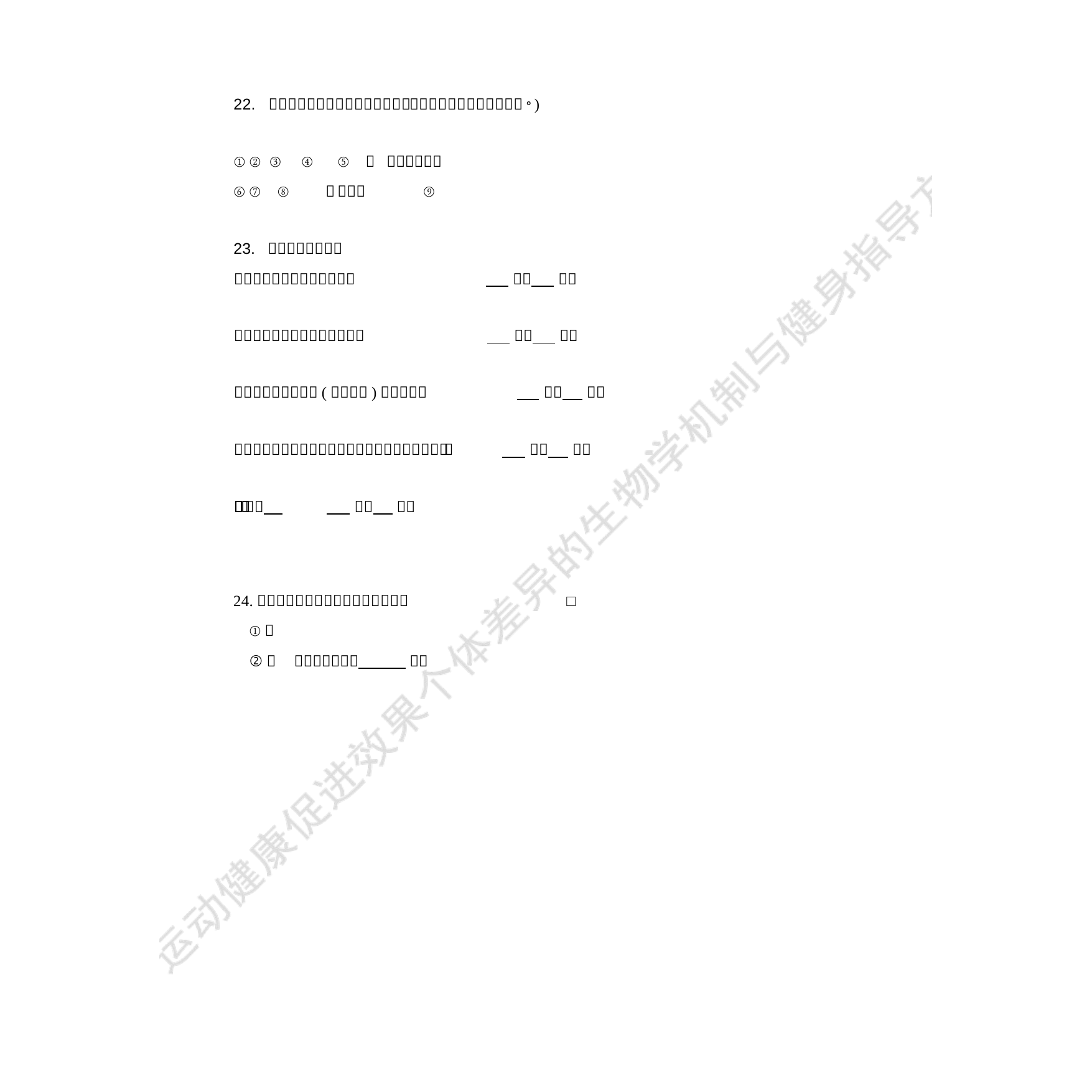

22. 您不参加运动健身的原因是什么？（如果您已参加，不回答此题。此题可多项选择)
①不喜欢 ②无时间 ③无场地器材 ④身体状况不适合 ⑤经济能力不足
⑥缺少技术指导 ⑦缺乏运动同伴 ⑧怕被笑 ⑨其他
23. 静态活动行为调查
您平均每天看电视的时间是： 小时 分钟
您平均每天使用电脑的时间是： 小时 分钟
您平均每天用于阅读(纸质书籍)的时间是： 小时 分钟
您平均每天玩电子游戏的时间是（不包括电脑游戏）： 小时 分钟
其他以坐姿为主的活动（不包括睡眠、吃饭） 如： 小时 分钟
24.（此问题限女性回答）是否已经闭经 □
①否
②是 闭经时的年龄为 周岁

## Slide 8
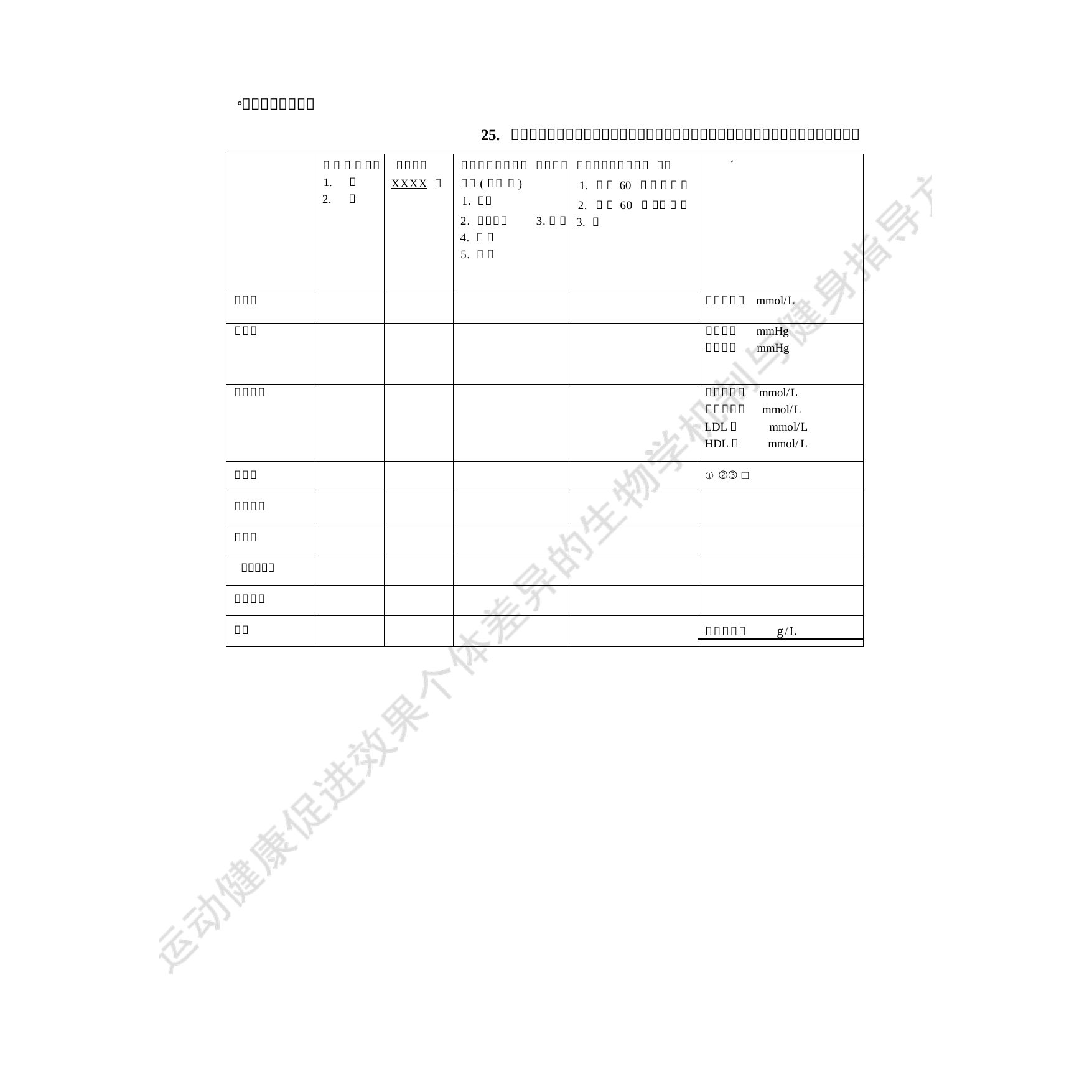

为了更为详尽了解您的健康状况，请您耐心填写下表。如有疑问，请与研究人员沟通！
25. 您是否被医生告知患有下列疾病？如果有，请填写患病时间、家族史、化验结果等信息。
| | 是 否 患 有 该病 1. 是 2. 否 | 患病时间 XXXX 年 | 您采取了什么措施 来治疗疾病？(可多 选) 1. 服药 2. 控制饮食 3.运 动 4. 手 术 5. 其 他 | 您的父母是否患过该 病？ 1. 是 ， 60 岁 以 下 发 病 2. 是 ， 60 岁 以上 发 病 3. 否 | 近期化验、检测情况 |
| --- | --- | --- | --- | --- | --- |
| 糖尿病 | | | | | 空腹血糖： mmol/L |
| 高血压 | | | | | 收缩压： mmHg 舒张压： mmHg |
| 血脂异常 | | | | | 甘油三酯： mmol/L 总胆固醇： mmol/L LDL： mmol/L HDL： mmol/L |
| 脂肪肝 | | | | | ①轻度②中度③重度： □ |
| 骨质疏松 | | | | | |
| 冠心病 | | | | | |
| （脑）卒中 | | | | | |
| 骨关节炎 | | | | | |
| 贫血 | | | | | 血红蛋白： g/L |
| | | | | | |

## Slide 9
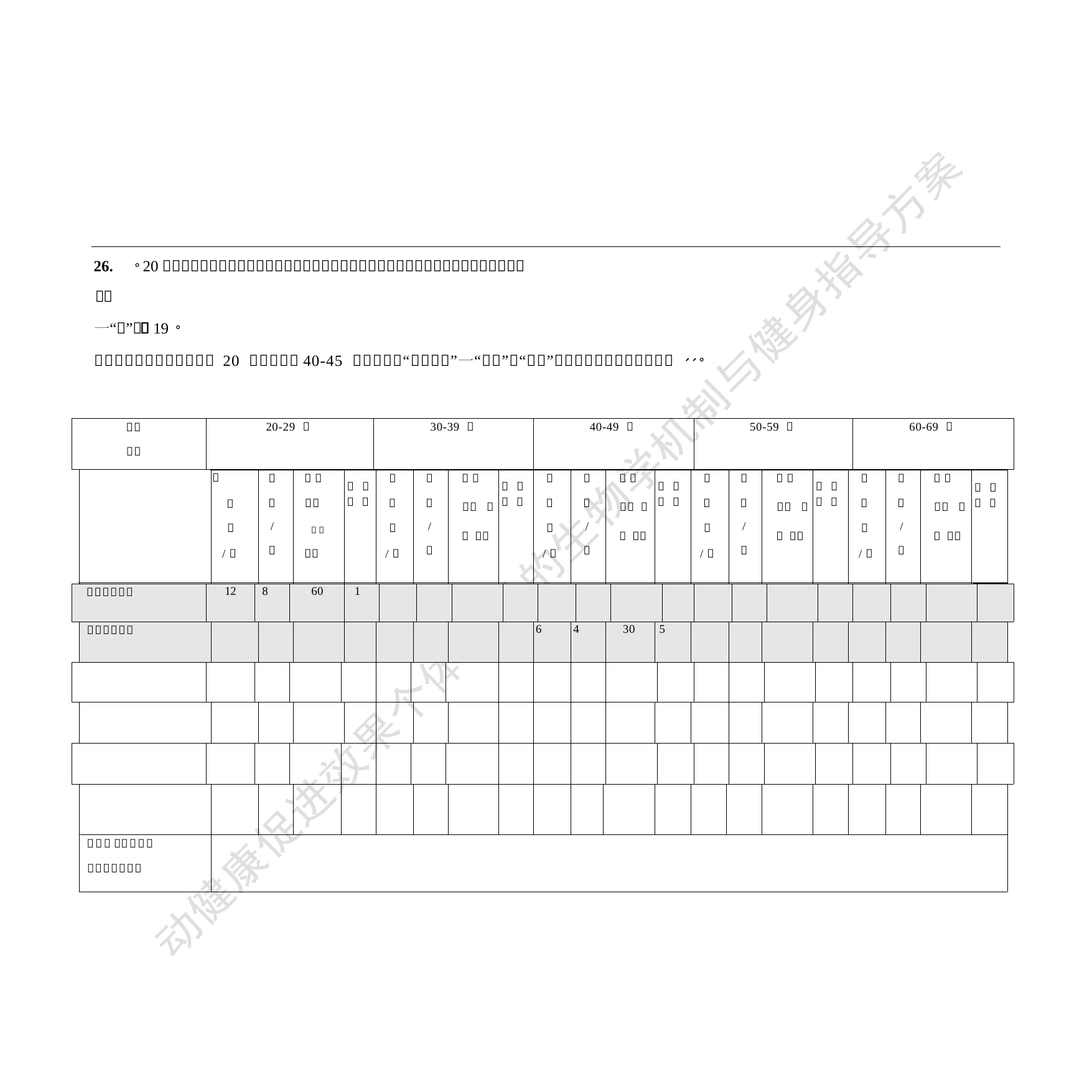

26. 既往体育锻炼情况调查。请您回忆自20岁以来，是否阶段性从事过较为规律的体育锻炼？曾经练习过什么项目，练习的频率和时
间。
如果既往没有任何锻炼的经历，请在锻炼项目一栏中注明“无”。如您已在前面（19题）填过的体育锻炼信息不要在这里重复填写。
填表举例说明：如果您曾在 20 岁打排球，40-45 岁游泳，在“锻炼项目”一栏内填写“排球”和“游泳”，同时在这两个年龄段对应 的表格内填上练习的次数、练习的时间、练了多少年，见下表举例说明。
| 锻炼 项目 | | 20-29 岁 | | | | | | | | 30-39 岁 | | | | | | | | | | | 40-49 岁 | | | | | | | | | | | 50-59 岁 | | | | | | | | | | | 60-69 岁 | | | | | | | |
| --- | --- | --- | --- | --- | --- | --- | --- | --- | --- | --- | --- | --- | --- | --- | --- | --- | --- | --- | --- | --- | --- | --- | --- | --- | --- | --- | --- | --- | --- | --- | --- | --- | --- | --- | --- | --- | --- | --- | --- | --- | --- | --- | --- | --- | --- | --- | --- | --- | --- | --- |
| | | | 几 个 月 /年 | | 次 数 / 月 | | 每 次 时间 （ 分 钟） | | 坚 持 年 限 | | 几 个 月 /年 | | | 次 数 / 月 | | | 每 次 时间 （ 分 钟） | | 坚 持 年 限 | | 几 个 月 /年 | | 次 数 / 月 | | | 每 次 时间 （ 分 钟） | | 坚 持 年 限 | | | 几 个 月 /年 | | | 次 数 / 月 | | 每 次 时间 （ 分 钟） | | | 坚 持 年 限 | | | 几 个 月 /年 | | 次 数 / 月 | | 每 次 时间 （ 分 钟） | | 坚 持 年 限 | | |
| 排球（举例） | | 12 | | 8 | | 60 | | | 1 | | | | | | | | | | | | | | | | | | | | | | | | | | | | | | | | | | | | | | | | | |
| | 游泳（举例） | | | | | | | | | | | | | | | | | | | | 6 | | 4 | | | 30 | | 5 | | | | | | | | | | | | | | | | | | | | | | |
| | | | | | | | | | | | | | | | | | | | | | | | | | | | | | | | | | | | | | | | | | | | | | | | | | | |
| | | | | | | | | | | | | | | | | | | | | | | | | | | | | | | | | | | | | | | | | | | | | | | | | | | |
| | | | | | | | | | | | | | | | | | | | | | | | | | | | | | | | | | | | | | | | | | | | | | | | | | | |
| | | | | | | | | | | | | | | | | | | | | | | | | | | | | | | | | | | | | | | | | | | | | | | | | | | |
| | 备注： （对上述填 写内容做说明） | | | | | | | | | | | | | | | | | | | | | | | | | | | | | | | | | | | | | | | | | | | | | | | | | |

## Slide 10
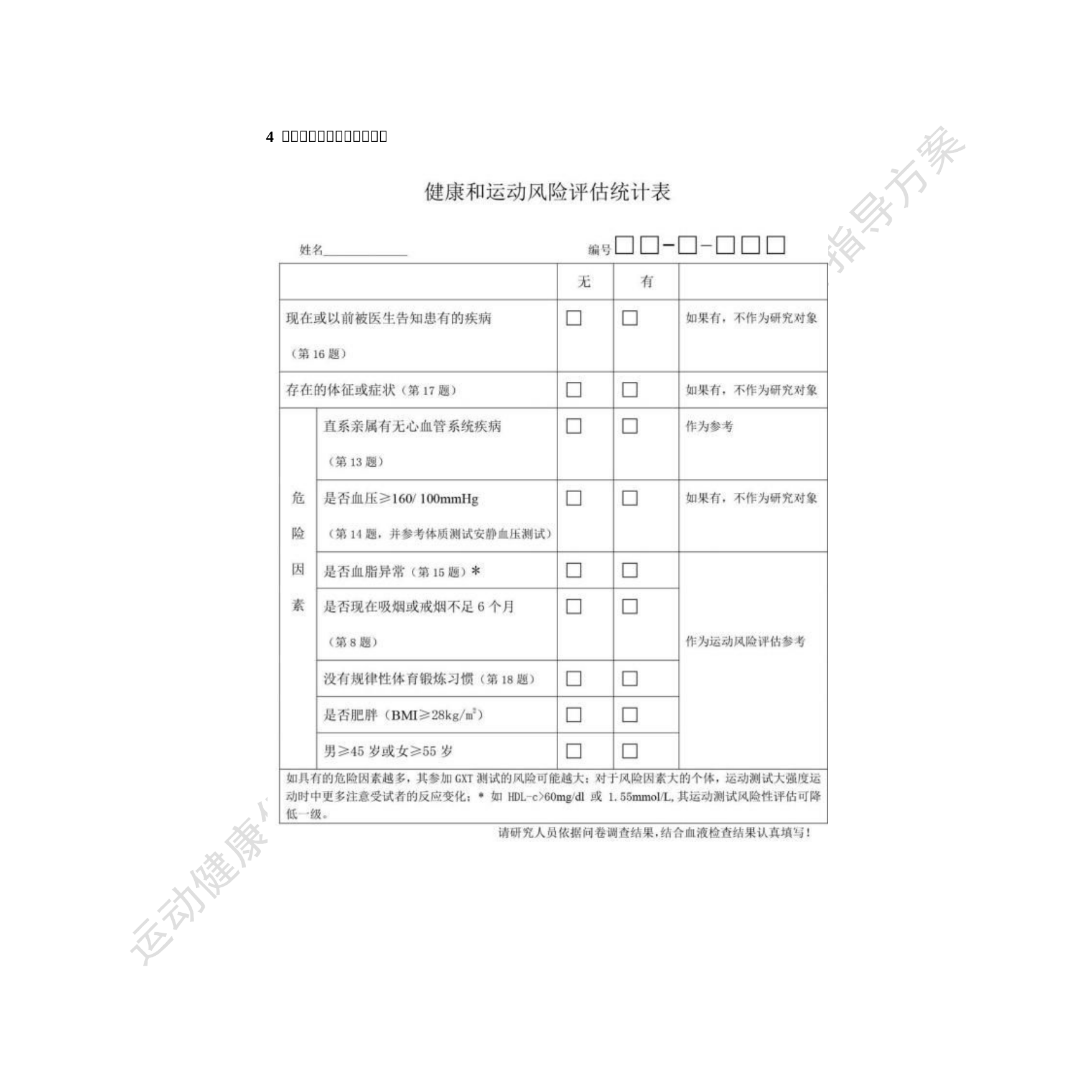

4 健康和运动风险评估统计表
尺

## Slide 11
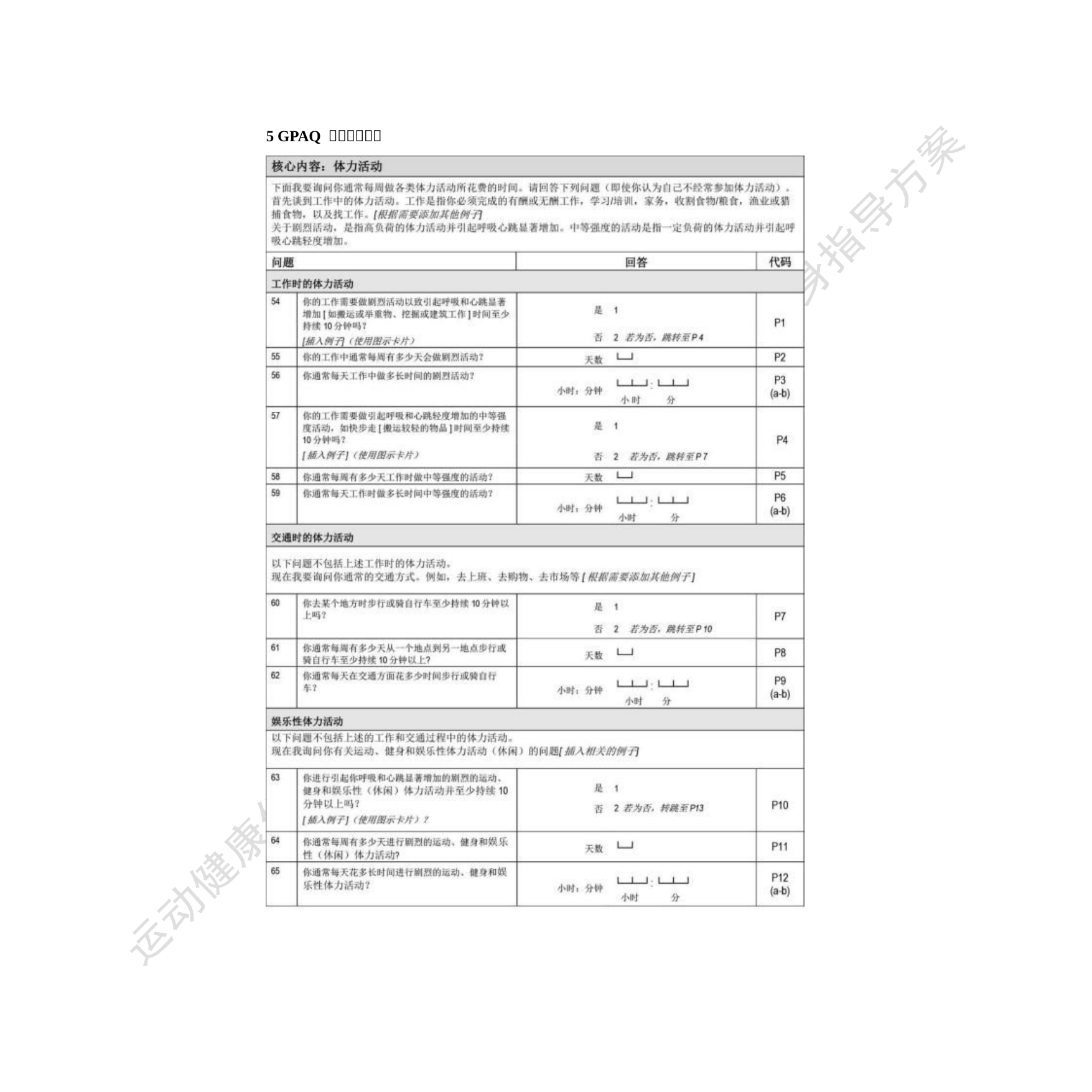

5 GPAQ 体力活动问卷

## Slide 12
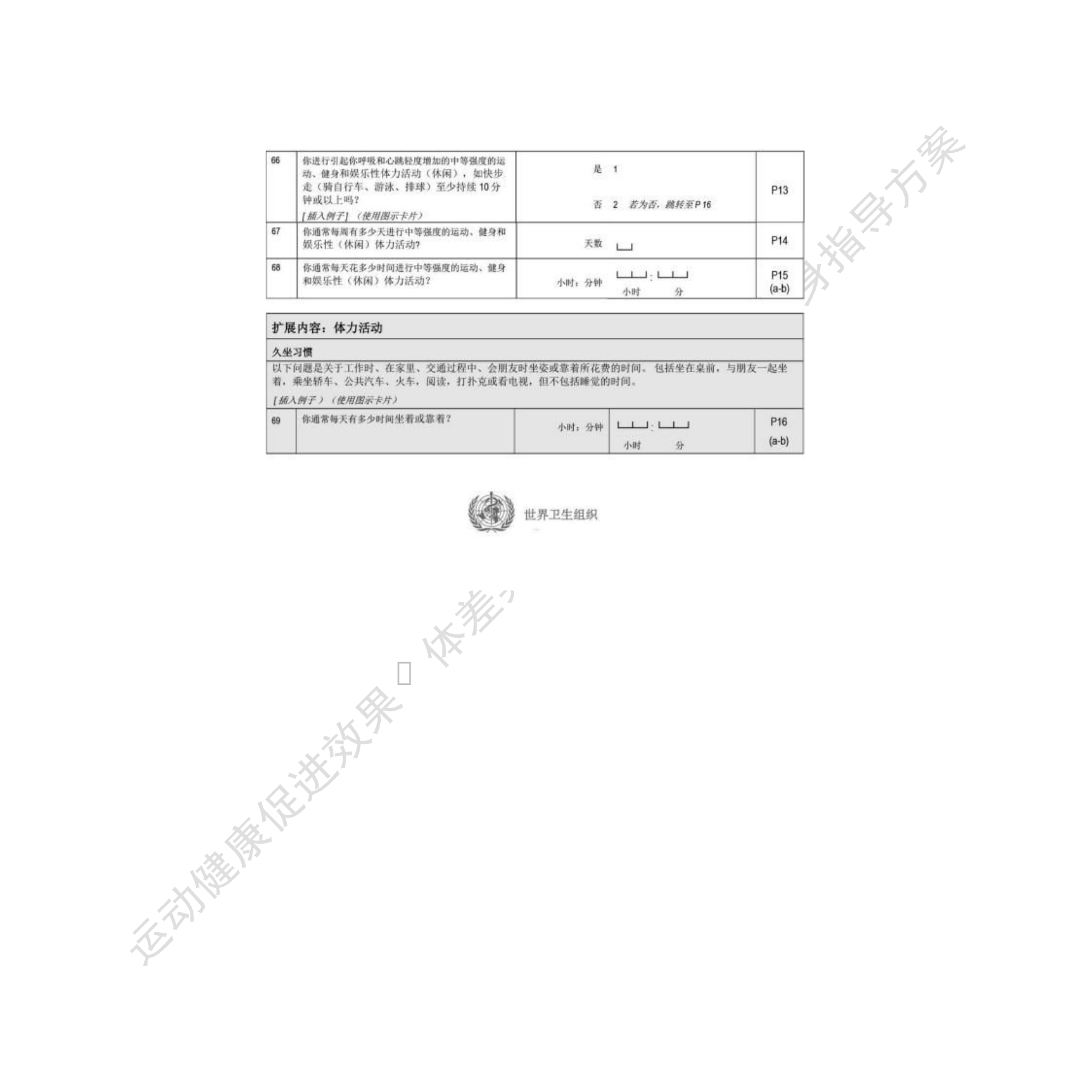

尺

## Slide 13
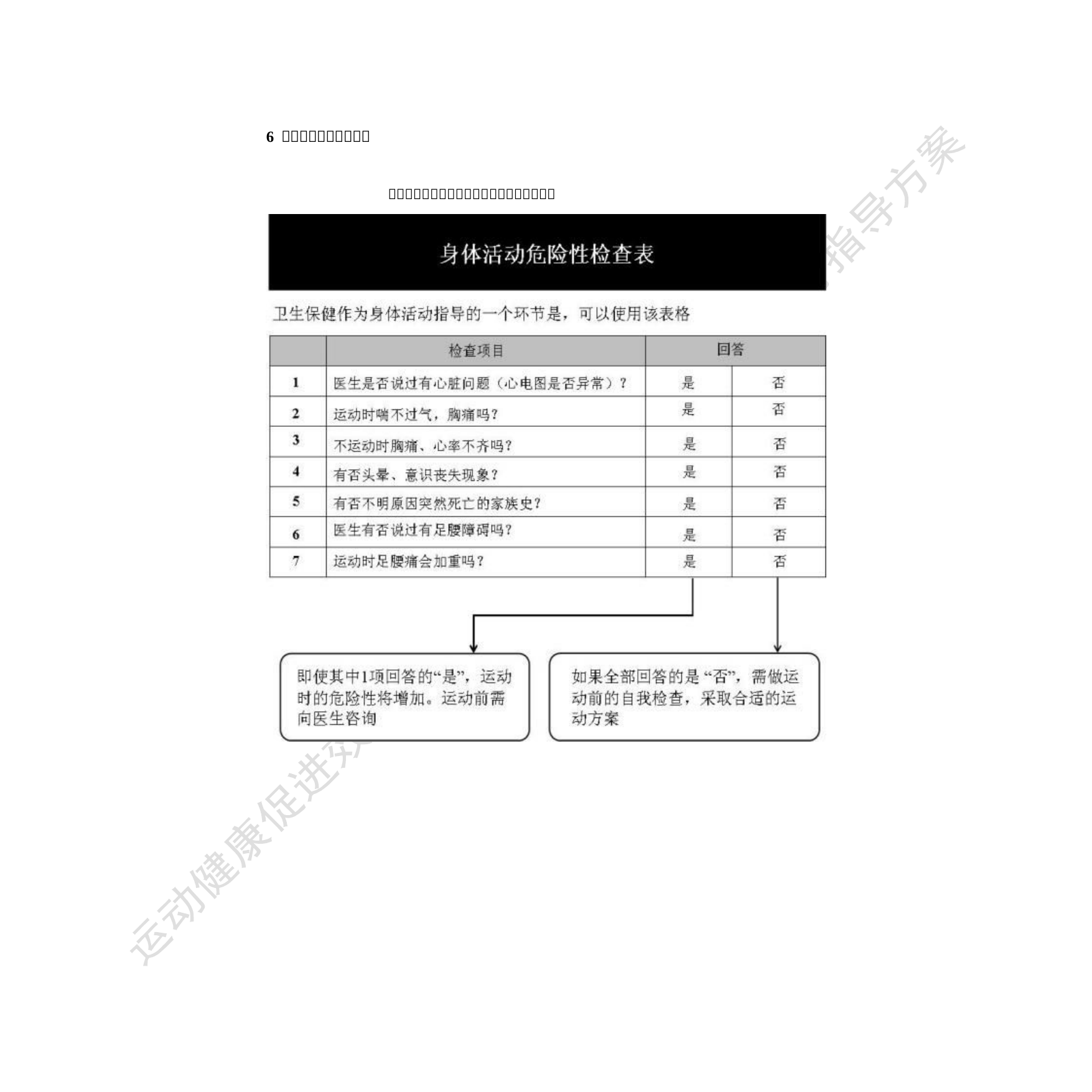

6 身体活动危险性检查表
（此表用于每次运动前身体状况的自我评估）

## Slide 14
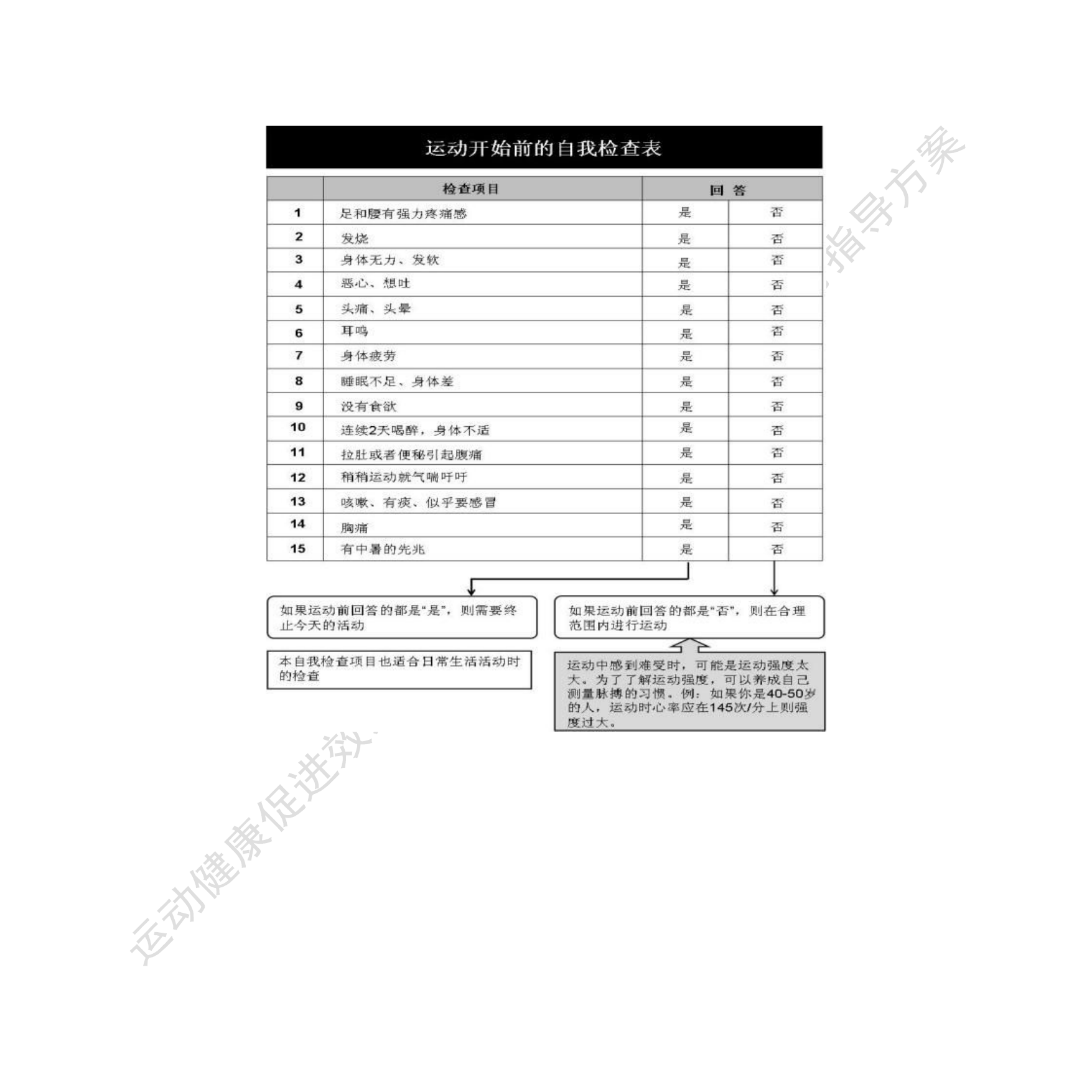

Supplement: Supplemental Information 4 [file peerj-13-19271-s004.pptx]
